# Genetic manipulation of the interconversion between diacylglycerols and triacylglycerols in *Rhodosporidium toruloides*

**DOI:** 10.3389/fbioe.2022.1034972

**Published:** 2022-10-26

**Authors:** Yue Zhang, Sufang Zhang, Yadong Chu, Qi Zhang, Renhui Zhou, Di Yu, Shuang Wang, Liting Lyu, Guowang Xu, Zongbao Kent Zhao

**Affiliations:** ^1^ Laboratory of Biotechnology, Dalian Institute of Chemical Physics, CAS, Dalian, China; ^2^ University of Chinese Academy of Sciences, Beijing, China; ^3^ Dalian Key Laboratory of Energy Biotechnology, Dalian Institute of Chemical Physics, Chinese Academy of Sciences, Dalian, China

**Keywords:** oleaginous yeast, RNA interference, lipid synthesis, diacylglycerol, lipase, synthetic biology

## Abstract

The basidiomycetous yeast *Rhodosporidium toruloides* (*R. toruloides*) is an excellent producer for neutral lipids, including triacylglycerols (TAG). Partially because genetic tools for this yeast were less developed, limited efforts were shown to explore its capacity for the production of higher-value lipids such as diacylglycerols (DAG). Here, four genes linked to the interconversion between DAG and TAG were manipulated to promote the production of DAG and free fatty acids (FFA). Among them, three TAG synthesis-related genes, *DGA1*, *LRO1,* and *ARE1*, were down-regulated successively *via* the RNA interference technology, and an endogenous TAG lipase encoded by *TGL5* was fused with *LDP1* and over-expressed to convert TAG into DAG and FFA. Results showed that those engineered *R. toruloides* strains grew normally under nutrient-rich conditions but notably slower than the parental strain NP11 in the lipid production stage. When cultivated in nitrogen-limited media, engineered strains were able to produce total lipids with improved contents of DAG and FFA by up to two-fold and three-fold, respectively. Further correlation analysis between lipid composition and cell density indicated that the formation of TAG correlated positively with cell growth; however, other lipids including DAG did negatively. This study offered valuable information and strains to engineer *R. toruloides* for advanced production of fatty acid derivatives.

## Introduction

Lipids especially vegetable oils are important primary commodities for the production of foods, oleochemicals, and biofuels. While vegetable oils are traditionally produced by plants and animals, the capacity of the traditional approaches is limited in terms of quality and quantity to match the ever-growing market demands (Szczepanska et al., 2022). In the past two decades, much attention from academia and industry has been paid to lipids from heterotrophic microorganisms, partially because the production of lipids under controlled conditions offers several advantages. These include the utilization of cheap raw materials like lignocellulosic biomass for large-volume production, the requirement of less arable land and forests, accessibility to diverse lipid molecules with engineered hosts, and better compatibility with other continuous industrial processes (Wang et al., 2021).

Microorganisms that can accumulate lipids to more than 20% of their cell mass are considered oleaginous. There are several genera of oleaginous fungi, such as *Yarrowia, Rhodotorula, Lipomyces* and *Mortieralla*. In general, lipids produced by oleaginous yeasts resemble vegetable oils being commonly used in terms of triacylglycerols (TAG) contents and fatty acid compositional profiles (Szczepanska et al., 2022; Uprety et al., 2022). The basidiomycetous fungus *Rhodosporidium toruloides* (synonym *Rhodotorula toruloides*), is naturally an oleaginous yeast that can accumulate lipids to more than 70% of its dry cell weight and can use diverse carbon sources (Li et al., 2007). In addition, *R. toruloides* can naturally produce carotenoids and valuable metabolites, which makes it an attractive workhorse for various biotechnological applications (Park et al., 2018; Wen et al., 2020). Over the past decade or so, systems biology studies including genomics, transcriptomics, proteomics, and genome-scale metabolic modeling have been performed, leading to advanced understanding in terms of the fundamentals behind the phenotypic uniqueness of *R. toruloides* (Zhu et al., 2012; Coradetti et al., 2018; Wang et al., 2018). Meanwhile, transformation methods (Lin et al., 2014; Liu et al., 2017) and genetic components including promoters, terminators and selection markers (Wang et al., 2016; Nora et al., 2019) have been documented to engineer *R. toruloides*. As a result, engineered *R. toruloides* strains have demonstrated improved profiles in terms of stress resistance, product portfolio, and production capacity.

Diacylglycerols (DAG) are valuable lipids as anti-obesity functional oils for better bone health. In particular, *sn*-1,3-DAG demonstrated great potential in suppressing body fat accumulation and lowering postprandial serum TAG, cholesterol, and glucose levels (Lee et al., 2020). Because DAG is present only as a trace component in vegetable oil products, some efforts have been devoted to converting TAG into DAG by using different lipases (Wang et al., 2012; Huang et al., 2022). It is known that DAG is an important biosynthetic precursor to TAG; however, DAG also serves as a second messenger of intracellular lipid metabolism and a precursor for the synthesis of cell membrane components such as glycerophospholipids (Eichmann and Lass., 2015). Considering its complicated cellular roles, microbial production of DAG *via* metabolic engineering has been rarely reported (Eichmann and Lass, 2015). Nonetheless, it remains interesting to investigate whether oleaginous yeasts can be engineered to accumulate DAG in large quantities because in principle DAG could be stored in bulky lipid droplets (LDs) found in oleaginous yeasts.

Here, we knocked down three genes of *R. toruloides* NP11 related to the conversion of DAG into TAG, namely, *DGA1*, *LRO1* and *ARE1*, encoding diacylglycerol acyltransferase (DGAT), phospholipid: DAG acyltransferase (PDAT) and acyl-CoA: sterol acyltransferase (ACAT), respectively. Subsequently, the endogenous gene *TGL5* encoding a native TAG lipase was overexpressed in-fusion with lipid droplet protein Ldp1 to facilitate DAG formation *via* TAG hydrolysis. It was found that those engineered strains grew normally under nutrient-rich conditions but substantially slower than the parental strain during the lipid production phase. Importantly, total lipids with improved DAG contents by up to two-fold were produced by those strains grown in nitrogen-limited media. More detailed analysis indicated that the cell growth of *R. toruloides* variants correlated positively with the content of TAG but negatively with the contents of other lipids, including DAG and free fatty acids (FFA). Our results suggested that the metabolic reactions involved in TAG are relatively flexible to genetic intervention in the oleaginous yeast *R. toruloides*.

## Materials and methods

### Microbial strains, media and general growth conditions

The haploid strain *R. toruloides* NP11 (MAT A1) was accessible from the Guangdong Microbial Culture Collection Center (GDMCC 2.224). *Escherichia coli* DH5α, *E. coli* DH10B and *Agrobacterium tumefaciens* AGL1 were lab collection strains. Additional information for microbial strains is listed in Table 1.

**TABLE 1:** Strains and plasmids were used in this study.

Strains and plasmids	Genotype or characteristic	Origin
Strains		
*R. toruloides* NP11	MAT A	Zhu et al. (2012)
*R. toruloides* LROi815	NP11 with genome-integrated LROi cassette	This study
*R. toruloides* DLi2	NP11 with genome-integrated DGAi-LROi cassette	This study
*R. toruloides* DLAi12	NP11 with genome-integrated DGAi-LROi and ARE1i cassette	This study
*R. toruloides* TGL544	NP11 with genome-integrated *TGL5-LDP1* cassette	This study
*R. toruloides* DLiT223	DLi2 with genome-integrated *TGL5-LDP1* cassette	This study
*R. toruloides* DLAiT1226	DLAi12 with genome-integrated *TGL5-LDP1* cassette	This study
*Escherichia coli* DH5α	*Escherichia coli F* ^−^, *φ80dlacZΔM15, Δ(lacZYA-argF)U169*, *deoR*, *recA1*, *endA1*, *hsdR17(rK* ^*-*^ *, mK* ^*+*^ *)*, *phoA*, *supE44*, *λ* ^*-*^, *thi-1*, *gyrA96*, *relA1*	Takara
*Escherichia coli* DH10B	*Escherichia coli F- endA1 recA1 galU galK deoR nupG rpsL ΔlacX74 φ80lacZΔM15 araD139 Δ(ara, leu) 7697 mcrA Δ(mrr-hsdRMS-mcrBC) λ-*	Invitrogen
*Agrobacterium tumefaciens* AGL1	AGL0 *recA::bla* pTiBo542DT Mop ^+^ CbR	Lazo et al. (1991)
Plasmids		
pZPK-MCS	pZPK-P_*PGK*_-HYG-T_*NOS*_-P_*GPD*_-MCS-T_*HSP*_	Lab Collection
pZPK-LRO1i	pZPK-P_*PGK*_-HYG-T_*NOS*_-P_*GPD*_-LRO1i-T_*HSP*_	This study
pZPK-DGA1i-LRO1i	pZPK-P_*PGK*_-HYG-T_*NOS*_-P_*GPD*_-DGA1i/LRO1i-T_*HSP*_	This study
pZPK-ARE1i	pZPK-P_*PGK*_-NAT-T_*NOS*_-P_*GPD*_-ARE1i-T_*HSP*_	This study
pZPK-TGL5-LDP1	pZPK-P_*PGK*_-TGL5-LDP1-F2A-Ble-T_*HSP*_	This study

*E. coli* DH5α/DH10B and *A. tumefaciens* AGL1 cells were cultivated in Luria-Bertani (LB) medium containing tryptone (10 g/L), yeast extract (5 g/L), and NaCl (10 g/L), initial pH 7.0 in a 200-rpm shaker at 37°C and 30°C, respectively. *R. toruloides* cells were cultivated in YPD medium containing glucose (20 g/L), yeast extract (10 g/L), peptone (20 g/L), initial pH of 6.0 at 30°C. For lipid production, *R. toruloides* cells were pre-cultured in a 250-ml Erlenmeyer flask with 50 ml working volume at 30°C for 24 h. Subsequently, cells were inoculated into nitrogen-limited (NL) medium containing the following components per liter: glucose·H_2_O 70 g, yeast extract 0.75 g, (NH_4_)_2_SO_4_ 0.1 g, KH_2_PO_4_ 1.0 g, MgSO_4_·7H_2_O 1.5 g, and 10 ml of trace element solution, which contains per liter: CaCl_2_·2H_2_O 4.0 g, FeSO_4_·7H_2_O 0.55 g, citric acid·H_2_O 0.52 g, ZnSO_4_·7H_2_O 0.10 g, MnSO_4_·H_2_O 0.076 g and 100 μl 18 M H_2_SO_4_, in 50 mM 2-morpholinoethanesulphonate (MES) buffer (pH 6.0). All media were sterilized at 121°C for 20 min.

### Primers, plasmids and strains construction

**FIGURE 1:**
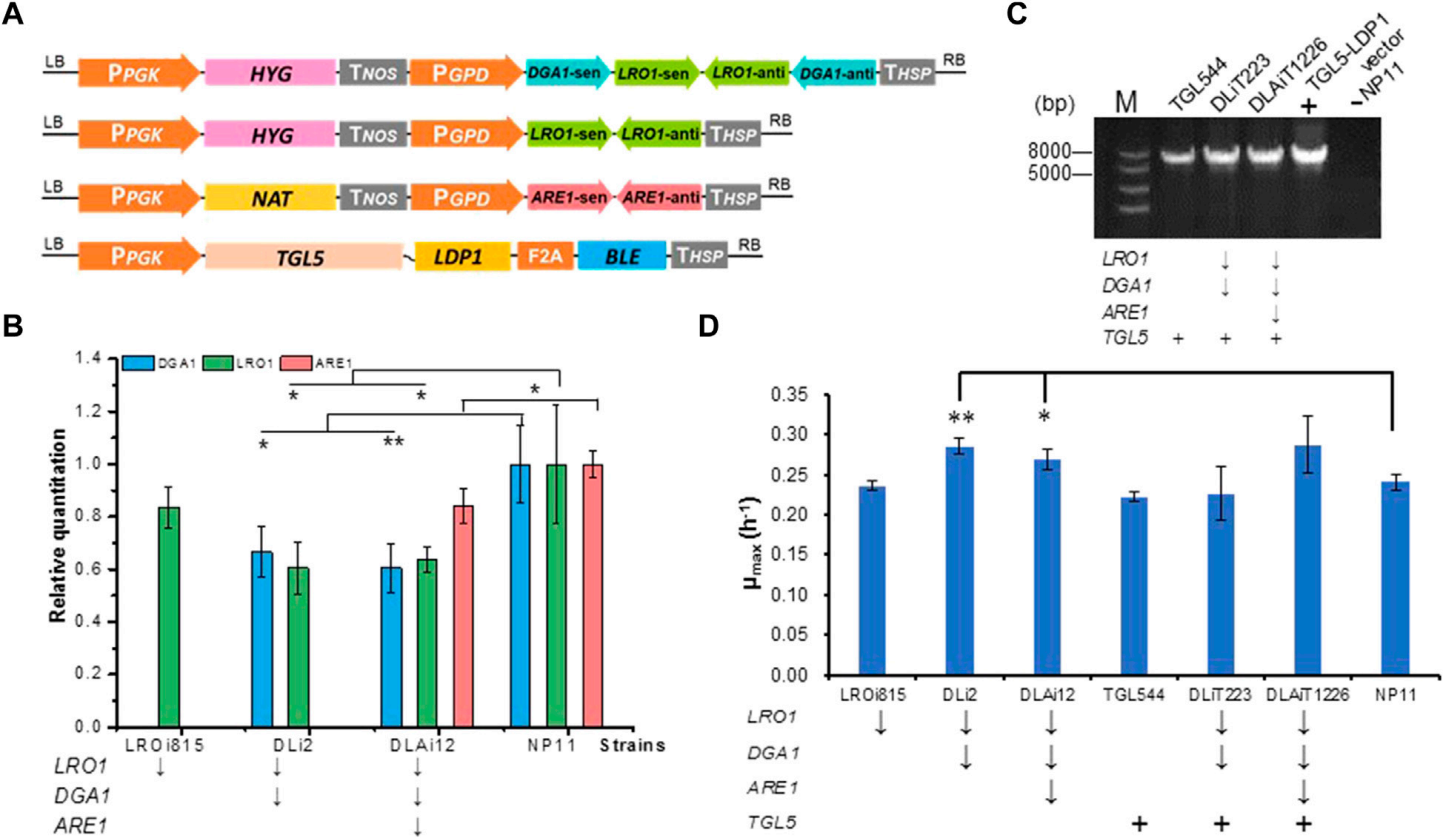
Construction and verification of *R. toruloides* variants. **(A)** Diagram of RNAi plasmids and the *TGL5*-*LDP1* genes overexpression plasmid. Abbreviations: HYG, Hygromycin gene; NAT, Nourseothricin acetyltransferase gene; BLE, Bleomycin gene. **(B)** *DGA1, LRO1,* and *ARE1* expression levels of different RNAi engineering strains by RT-qPCR. *DGA1*, diacylglycerol acyltransferase; *LRO1*, phospholipid diacylglycerol acyltransferase; *ARE1*, acyl-CoA sterol acyltransferase; RT-qPCR, reverse transcription quantitative polymerase chain reaction. **p < 0.01, *p < 0.05 of one-way factor AVONA. **(C)** The verification of *TGL5-LDP1* genes overexpression in engineered strains. Agarose gel (1%) electrophoresis map of *TGL5-LDP1* (5670 bp) by PCR amplification from promoter P_PGK_ (from 777 sites) to BLE (26 sites). **(D)** Maximum specific growth rate (μ_max_) of strains in YPD medium. The gene labeled with ↓ was knocked down, with + was overexpressed.

The primers used for fragments and the plasmids used for strain construction are listed in Supplementary Table S1 and Table 1, respectively. All DNA fragments obtained by polymerase chain reaction (PCR) were gel purified by using a kit (Sangon Biotech; Shanghai, China) before further applications. Vectors used for one-gene RNAi were constructed according to the protocols described previously (Liu et al., 2019). For two-gene simultaneous RNAi (Figure 1A), vectors were constructed as follows, two genes reverse complementary oriented fragments were PCR amplified, fused by recombinant PCR, and finally ligated the segments into the plasmid restriction site using the In-Fusion HD Cloning Kit (Takara, Dalian, China). The vector for expression of *TGL5*-*LDP1* fusion-expression was constructed based on the method of co-expression of multiple enzymes from a single promoter mediated by virus 2A sequence (F2A) (Jiao et al., 2018). All genes were under the control of constitutive promoters (P_*PGK*_ or P_*GPD*_). Vectors for RNAi and protein expression were electro-transformed into *A. tumefaciens* AGL1 competent cells. Then *R. toruloides* variants were constructed by *Agrobacterium tumefaciens-*mediated transformation (ATMT) based on a published method (Lin et al., 2014).

### Genotypic analysis of *R. toruloides* transformants

Genotypes of the *R. toruloides* transformants were verified by using the colony PCR method (Lin et al., 2012). Total RNA isolation and RT-qPCR analysis were done according to procedures described elsewhere (Liu et al., 2019), and reverse transcription was performed by using the PrimeScript RT reagent Kit (Takara, Japan). Quantitative PCR was performed by using SYBR *Premix Ex Taq* (TaKaRa, Japan) on a Real-Time Quantitative PCR instrument (Light Cycler 96, Roche, Sweden or Eco Real-Time PCR System, Illumina, United States). Data were analyzed using the relative quantitation/comparative threshold cycle (ΔΔCT) method (Tanic et al., 2007) and were normalized to the endogenous gene ACTIN.

### Lipid production culture

The strains were activated on the YPD plates at 28°C for 72 h. For seed culture preparation, a loop of the activated cells were cultivated in 250-ml shaking flasks with 30 ml of liquid YPD media at 30°C, 200 rpm for 24 h. Subsequently, 250-ml Erlenmeyer flasks contained 45 ml of nitrogen-limited medium (70 g/L glucose as above media, pH 6.0) were inoculated with 5 ml of the seed culture, and held at 30°C, at 200 rpm until the residual glucose dropped below 10 g/L. During the culture, the residual glucose was monitored offline by the SBA-50E glucose analyzer (Shandong Academy of Sciences, China) every 24 h.

### Lipid extraction procedures for compositional analysis

To avoid changes in intracellular lipid compositions, a rapid quenching method was used according to the literature (Canelas et al., 2008) with minor modifications. Briefly, about 2.5*10^7^ yeast cells (nearly 25 OD_600_) into an Eppendorf tube were mixed thoroughly with 1 ml of pre-cooled (−20°C) 60% aqueous methanol (v/v) solution for 5 s, centrifuged at 4000 *g* for 5 min at −20°C, followed by lipid extraction immediately or treated with liquid nitrogen and then kept at −80°C until lipid extraction.

Cells were ruptured with glass beads by using FastPrep equipment (Fast Prep-24, MP Biomedicals). Briefly, cells in 500 μl of PBS buffer (0.01 M) and 0.5 g of glass beads (20 μm) were resuspended in 2-ml screw-cap tubes, shaken for 60 s at the speed of 4.0 M/s for two cycles, set on ice-bath for 1 min, and then treated for additional two cycles. After cell rupture, 300 µl of supernatants were transferred into a 2-ml tube for lipid extraction. According to the Bligh and Dyer method (Bligh and Dyer., 1959), 450 µl of chloroform and 450 μl of methanol were added to the tubes, vortexed for 1 min, centrifuged at 8000 *g* for 5 min, and the lower layer transferred to a 7-ml tube. Recover 450 μl of chloroform and repeat the operation twice or three times until the chloroform phase is clear. Then an equal volume of 0.1% NaCl solution was added, vortexed for 1 min, centrifuged at 8000 *g* for 5 min, and the recovered chloroform phase was dried with anhydrous sodium sulfate. Samples were treated by rotary evaporation, held at 35°C for 5 min, transferred to a 2-ml sample bottle upon adding chloroform to a final volume of 1 ml, and then stored at −20°C before HPTLC analysis.

### High-performance thin-layer chromatography (HPTLC) analysis of neutral lipids

The lipid composition analysis was performed by the high-performance thin-layer chromatography method (HPTLC) (Olsen and Henderson., 1989) with modifications, and the relative lipid quantification was evaluated with HPTLC Densitometer (Biostep-CD60, DESAGA, German). Precoated HPTLC silica gel 60 plates without a fluorescent indicator (20*10 cm) were acquired from E. Merck (Darmstadt, Germany). The purity of lipid standards, including triacylglycerols (TAG, Acros, Belgium), diacylglycerols (DAG contains 1,2-DAG and 1,3-DAG, Macklin, China), monoglyceride (MAG), 2-stearoylglycerol (2-MAG, Sigma, Germany), sphingomyelin (SM, Sigma, Germany) and oleic acid (free fatty acid, FFA, Aladdin, China) was ≥99% (GC). Add 5 μL lipid samples and mixed standards to the plate using HPTLC Densitometer. The HPTLC plates were developed in n-hexane: diethyl ether: glacial acetic acid (80:40:1 by volume) to 10 cm from the origin for the separation of neutral lipids. After drying under a stream of hot air, the plates were sprayed with 10% (w/v) copper sulfate pentahydrate in 8% (v/v) phosphoric acid, followed by charring at 160°C for 4 min. Quantification of relative lipid components was performed at a wavelength of 700 nm using the TLC scanner Biostep-CD60 and analyzed with the HPTLC Densitometer software ProQuant V3.05. Lipids were qualitatively identified by the above-mentioned standards and their relative content was determined by the normalization method.

### Determination of cell mass and total lipids

Upon completion of lipid production culture, cells in 30 ml of culture broth were collected by centrifuging at 8000 *g* at 4°C for 5 min using pre-weighed 50 ml PP centrifugal tubes, washed twice with 50 vol% ethanol, dried in an oven at 105°C for 24 h to constant weight. Dry cell weight (DCW) was measured gravimetrically.

Total lipids were extracted by the classical acid heat method according to a previous report (Wu et al., 2011). The dried cells were digested with 4 M HCl (3 ml per 0.5 g dry cells) in a shaking water bath at 78°C, 200 rpm for 1 h. The total intracellular lipid was extracted three times with chloroform-methanol (1:1, v/v). The chloroform extracts were washed with 0.1% NaCl (w/v) solution and passed over an anhydrous Na_2_SO_4_ pad, the chloroform was then eliminated by reduced pressure rotary evaporation and the pre-weighed round bottom flasks containing the lipid concentrates were dried at 105°C to constant weight and measured the total lipid gravimetrically. The lipid titer was expressed in g/L (culture broth volume), and the lipid contents in weight percentage (wt%) were measured as total lipid produced per total dry cell weight. The lipid yield was calculated as gram lipid produced per gram consumed glucose (g/g consumed glucose), and the lipid productivity was calculated as lipid titer per day (g/L/d). The *t*-test was used for each metric of experimental data to see whether the properties of NP11 and variations were substantially different.

### Fatty acid composition analysis

The extracted total lipids were transformed into fatty acid methyl esters (FAMEs) according to a previously established method (Li et al., 2007), then analyzed by Gas Chromatography/Triple Quadrupole Mass Spectrometer (GC-MS, Agilent Technologies, 7890B-7000D) as reported (Yu et al., 2017) by using an HP-5MS column (30 m × 0.25 mm × 0.25 μm). Gaseous nitrogen was used as a carrier gas (1.1 ml/min). Sample (1 μl) was injected, and the split ratio was 1:100. Oven temperature was set at 80°C at the beginning for 1 min; increased to 230°C with a ramp rate of 80°C/min; then increased to 245°C with a ramp rate of 3°C/min, increased to 280°C with a ramp rate of 10°C/min, held for 5 min, post-run to 320°C, and held for 2 min. The temperatures of the MS transfer line and ion source were set at 250°C and 230°C, respectively. These setting conditions can detect all the short, medium, long, and very long-chain fatty acids of *R. toruloides*. FAMEs were qualitatively identified by standard samples and mass spectrometric ion peaks, and their relative content was determined by the area normalization method.

### Assessment of maximum specific growth rate

The maximum specific growth rate (μ_max_) of strains was studied in YPD media for 24 h as above description, and the OD_600_ was measured by the Bioscreen C instrument (Bioscreen C, Oy Growth Curves Ab Ltd., Finland) every 1 h. Initial OD_600_ was 0.1–0.2, and the fitting calculations of the μ_max_ value were calculated according to the method described by the literature (Hoffmann and Potter., 2002).

### Statistical analysis

One-way ANOVA was conducted to compare the NP11 group or control group with the engineering strains group by the analysis software. Data with *p* < 0.05 was considered statistically significant, *p* < 0.01 was considered statistically very significant, and *p* < 0.001 was considered statistically extremely significant.

Pearson correlation coefficient analysis was used to illustrate the correlation between the lipid components and cell growth (OD_600_), the calculation formula of Pearson product-moment correlation coefficient (r) is shown as follow $:r=\frac{\sum \left(x-\bar{x}\right)\left(y-\bar{y}\right)}{\sqrt{\sum {\left(x-\bar{x}\right)}^{2}\sum {\left(y-\bar{y}\right)}^{2}}}$ , x and y is the average of array1 (e.g. lipid content) and array 2 (e.g. OD_600_) respectively. When r > 0, there is a positive correlation between the two groups of variables; when r < 0, there is a negative correlation between the two groups of variables. The absolute value of r represents the strength of the correlation: |r| ≥ 0.75, the two variables can be considered highly correlated; 0.5 ≤ |r| < 0.7, the two variables can be considered moderately correlated; 0.3≤ |r| <0.5, the two variables can be considered lowly correlated; |r| <0.3, the two variables can be considered very weakly correlated or irrelevant.

## Results

### Identification of potential genes for the interconversion between DAG and TAG

Functional genes related to TAG synthesis and degradation in the yeasts *S. cerevisiae* and *Y. lipolytica* have been characterized over the years (Table 2). Based on this information and genome annotation data of *R. toruloides* NP11, we were able to identify *DGA1* gene (RHTO_01962) encoding acyl-CoA:diacylglycerol acyltransferase (DGAT) (Oelkers et al., 2002; Sorger and Daum., 2002; Athenstaedt, 2011) and *LRO1* gene (RHTO_01945) encoding phospholipid:diacylglycerol acyltransferase (PDAT) (Dahlqvist et al., 2000; Athenstaedt, 2011) for TAG biosynthesis. Moreover, *ARE1* gene (RHTO_00726) was selected because it encodes a protein homologous to the acyl-CoA:sterol acyltransferase (ACAT) in *S. cerevisiae* and DAG2 in *Y. lipolytica* (Supplementary Figure S1), both known with DAG acyltransferase activity (Beopoulos et al., 2012). Therefore, three native genes were suggested for the conversion of DAG into TAG in *R. toruloides*. It should be noted that the transcription of these genes (*DGA1, LRO1, ARE1*) were up-regulated under nitrogen limitation (Zhu et al., 2012) as well as inorganic phosphate (Pi) limitation (Wang et al., 2018) conditions that were used for lipid overproduction.

**TABLE 2:** TAG synthesis from DAG and hydrolysis-related enzymes in *S. cerevisiae* (SC), *Y. lipolytica* (YL), and *R. toruloides* (RT) NP11.

Gene	SC name	YL name	RT name	EC number	Enzyme function	Source
TAG synthesis from DAG						
*DGA1*	YOR245c	YALI0E32769g	RHTO_ 01962	EC 2.3.1.20	DGAT2, Acyl-CoA: diacylglycerol acyltransferase	(Athenstaedt, (2011); Oelkers et al. (2002); Sorger and Daum, (2002))
*LRO1*	YNR008w	YALI0E16797g	RHTO_ 01945	EC 2.3.1.158	PDAT, Phospholipid: diacylglycerol acyltransferase, has both phospholipase and acyltransferase functions and mediates the esterification of DAG, which uses phospholipids as acyl donors and DAG as acceptor	(Athenstaedt, (2011); Dahlqvist et al. (2000))
*ARE1*	YCR048w	YALI0F06578g	RHTO_ 00726	EC 2.3.1.26	ACAT, Acyl-CoA: sterol acyltransferase	(Oelkers et al. (2002); Sandager et al. (2002); Yazawa et al. (2012))
*ARE2*	YNR019w			EC 2.3.1.26	ACAT, Acyl-CoA: sterol acyltransferase	
TAG Hydrolase						
*TGL3*	YMR313c	YALI0D17534g	RHTO_ 03931	EC 3.1.1.3	ATGL, Triacylglycerol lipase	(Athenstaedt and Daum, (2003); Dulermo et al. (2013))
*TGL4*	YKR089c	YALI0F10010g		EC 3.1.1.3	Triacylglycerol lipase	(Athenstaedt and Daum, (2005); Dulermo et al. (2013))
*TGL5*	YOR081c			EC 3.1.1.3	Triacylglycerol lipase	

Triglyceride lipase (TGL) is predominantly responsible for the initial step of TAG hydrolysis, and TGL-overexpression can lead to the accumulation of DAG (Zimmermann et al., 2004). TGL associated with lipid droplets in eukaryotic cells exhibits the highest hydrolytic activity for the first ester bond of TAG but no detectable activity for other neutral lipids such as DAG and MAG (Zimmermann et al., 2004). Our previous analysis of the lipid droplet proteome of *R. toruloides* NP11 found that Tgl5p (RHTO_03931, *TGL5*) (Table 2) was associated with lipid droplets (Zhu et al., 2015). Indeed, the amino acid sequence of this Tgl5p contains the conserved motif GXSXG (^331^GTSAG^335^, Supplementary Figure S2) typical for lipolytic enzymes and has more than 30% sequence identity to the counterpart protein capable of hydrolyzing TAG in *S. cerevisiae* (Athenstaedt and Daum., 2003; Athenstaedt and Daum., 2005). Thus, *TGL5* was a potential candidate to be engineered for DAG formation.

**FIGURE 2:**
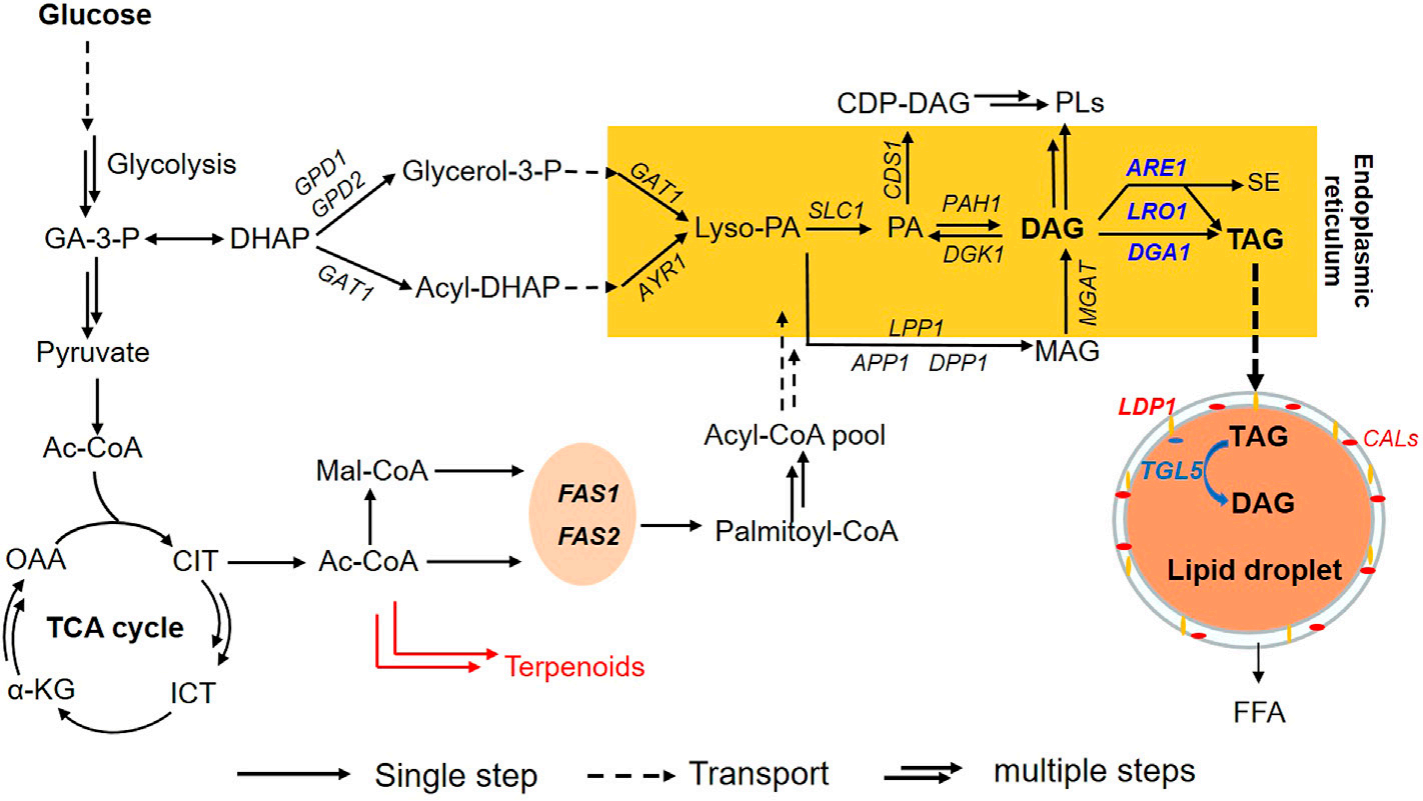
TAG related metabolism of *R. toruloides*. Metabolites (joined by arrows; a single arrow means one step, and double arrows mean multiple steps while a broken arrow means transport) and enzymes (named in the middle of the arrow) involved in TAG metabolism are shown. The commonly used names for metabolites and enzymes are used as abbreviations. Two cellular compartments for lipid synthesis and storage are also indicated: endoplasmic reticulum and lipid droplet, two important structural genes of lipid droplet were noted as yellow (*LDP1*) and red (*CALs*). Enzymes named in blue correspond to genes that have been already engineered in this study. *DGA1, LRO1, and ARE1* are three genes of the final step of TAG synthesis from DAG in *R. toruloides,* and *TGL5* is a TAG hydrolysis gene. Abbreviations: *FAS1* and *FAS2* are fatty acid synthetase; TAG, triacylglycerol; DAG, diacylglycerol; FFA, free fatty acid; MAG, monoglyceride; PL, phospholipids; PA, phosphatidic acid; CDP-DAG, cytidine diphosphate diacylglycerol; Lyso-PA, lysophosphatidic acid; SE, sterol ester.

Collectively, the TAG-related metabolism of *R. toruloides* was depicted in Figure 2. For lipid biosynthesis, DAG is converted into TAG *via* the enzymatic activities of *DGA1, LRO1* and *ARE1* in the endoplasmic reticulum (Zhu et al., 2012; Coradetti et al., 2018; Park et al., 2018). TAG is then mobilized into lipid droplets which are wrapped by structural proteins including Ldp1 and CALs (Zhu et al., 2015). Based on this information, we assumed that down-regulation of *DGA1*, *LRO1* and *ARE1*, in combination with overexpression of *TGL5* might lead to cells capable of accumulating lipids with substantially higher content of DAG.

### Genetic manipulation of endogenous genes related to DAG formation

To down-regulate the expression levels of endogenous genes in *R. toruloides*, we adopted the RNAi approach as described previously (Liu et al., 2019). Thus, RNAi plasmids were constructed to harbor expression cassettes for knocking down *LRO1* and *ARE1* individually or *DGA1* and *LRO1* simultaneously (Figure 1A; Table 1). Each expression cassette was integrated into the chromosome of the wild-type strain NP11 by *A. tumefaciens*-mediated transformation (Lin et al., 2014). For each experiment, 12 transformants were isolated and subjected to RT-PCR and RT-qPCR analysis. Results showed that the expression of these genes was indeed down-regulated (Figure 1B). A strain LROi815 was found with reduced expression levels of *LRO1* by 80%, and strain DLi2 showed reduced expression levels of *DGA1* (66%) and *LRO1* (57%). Subsequently, a strain DLAi12 was obtained upon the transformation of *R. toruloides* DLi2 with the *ARE1* downregulation cassette (Figure 1; Table 1). Compared to NP11, the down-regulation of those targeted genes was confirmed (Figure 1B).

To further promote DAG production, we overexpressed the native *TGL5* fused with *LDP1* to localize *TGL5* on lipid droplets for TAG hydrolysis. It should be noted that our previous study demonstrated that Ldp1 was an effective tag to localize partner proteins such as green fluorescent protein at the surface of lipid droplets (Zhu et al., 2015). A fusion-expression cassette encoding Tgl5p and Ldp1p (Tgl5-Ldp1) with bleomycin resistant marker linked by a 2A peptide (Jiao et al., 2018) was constructed (Figure 1A), and integrated into the genome of NP11, DLi2 and DLAi12, respectively, to give engineered strains TGL544, DLiT223 and DLAiT1226. Proper integration of the *TGL5* sequence was confirmed by PCR analysis of their genomic DNA samples (Figure 1C). Proper expression of Tgl5-Ldp1 was expected to warrant co-production of BLE for bleomycin resistance, and these engineered strains were selected in the presence of bleomycin. Collectively, *R. toruloides* strains of six different genotypes were obtained with an aim to produce lipids with higher contents of DAG (Table 1).

To test whether these genetic manipulations lead to growth defects, we cultivated *R. toruloides* variants in YPD media and monitored optical density changes at 600 nm every hour. The growth profiles of these engineered strains were found similar to that of the parental strain NP11. The maximal specific growth rates of the strains DLi2 and DLAi12 were even slightly higher than that of NP11 (Figure 1D). It was well-documented that oleaginous yeasts were unable to accumulate lipids when cultivated in nitrogen-rich media such as YPD media, implying that cells required minimal resources related to TAG metabolism. Therefore, it was reasonable to notice little difference in terms of cell growth among those strains with down-regulated expression of genes for TAG biosynthesis or the overexpressed gene for TAG hydrolysis.

### Lipid production profiles of the engineered strains

**FIGURE 3:**
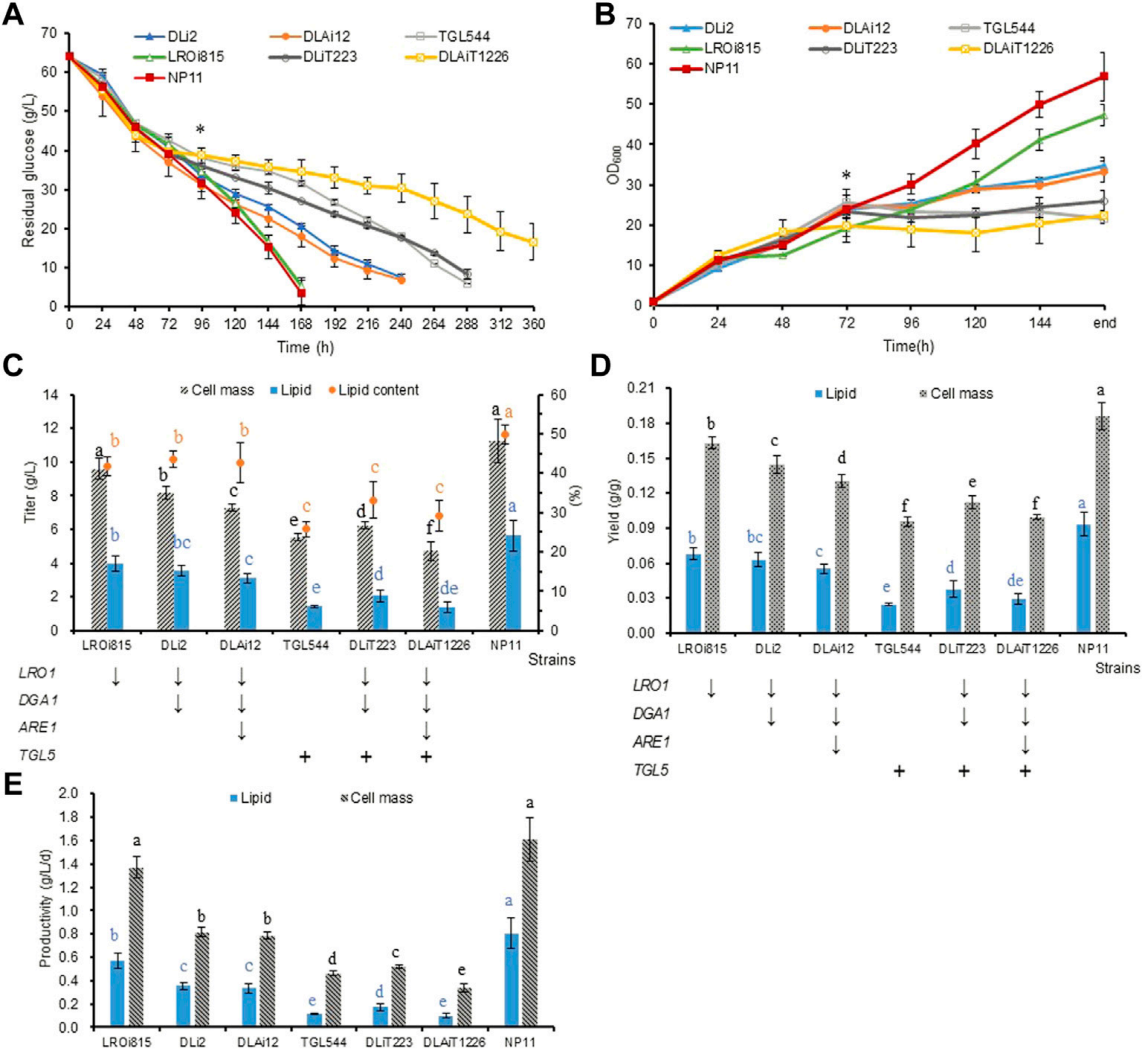
Lipid production profiles of *R. toruloides* variants under nitrogen limitation. **(A)** Profiles of glucose consumption. **(B)** Profiles of cell growth. End in Abscissa designated actual culture time of 168, 168, 240, 240, 288, 288, and 360 h for strains NP11, LROi815, DLi2, DLAi12, TGL544, DLiT223, and DLAiT1226, respectively, which were identical to those shown in **(A)**. **(C)** Results of cell mass, lipid titer, and total lipid contents. **(D)** Results of lipid and cell mass yields. **(E)** Results of lipid and cell mass productivity. Means without a common superscript letter and with * differ significantly (*p* < 0.05) as analyzed by the one-way factor AVONA.

To investigate the effects of these genetic manipulations on lipid production, we did shaking flask cultures of *R. toruloides* variants in NL media. It was found that glucose consumption and cell growth were nearly identical during the first 3 days, while differences were noticeable afterwards (Figures 3A,B). While LROi815 and NP11 shared similar glucose consumption profiles, the other five engineered strains assimilated glucose much slower, especially for the strain DLAiT1226. At the end of the culture, the optical cell densities of the strain TGL544 (21.5) and DLAiT1226 (22.4) were less than half of that of NP11 (56.9). These engineered strains had reduced titers of lipids and cell mass, reduced lipid contents (Figure 3C), lower yields of lipids and cell mass (Figure 3D), as well as reduced productivities (Figure 3E). These results suggested that *LRO1* plays an important role in TAG biosynthesis, which is in agreement with observations described elsewhere (Dahlqvist et al., 2000; Oelkers et al., 2000). For strains DLi2 and DLAi12 with Dga1 and Are1 being further downregulated, yields of lipids and cell mass reduced by 30–40%, and the lipid productivity decreased by more than 50%. Interestingly, there was no significant difference between DLi2 and DLAi12, except the cell mass yield of DLAi12 was a little lower than DLi2, indicating that the down-regulation of the *ARE1* gene had little influence on lipid accumulation*.* Based on the lipid production profiles of LROi815, DLi2, and DLAi12, it could be estimated that *DGA1* and *LRO1* play similar yet important roles in TAG synthesis, and *ARE1* is slightly inferior in *R. toruloides*. These results were consistent with results of lipid synthesis in *S. cerevisiae* (Sandager et al., 2002) and *Y. lipolytica* (Beopoulos et al., 2012). In addition, these features were further enhanced when the native lipase *Tgl5* was overexpressed. In the strain TGL544, the lipid and cell mass production decreased by 75% and 50%, respectively, and the engineered strains DLiT223 and DLAiT1226 did similarly (Figures 3C–E), suggesting that Tgl5 of *R. toruloides* has a strong hydrolysis effect on TAG as expected.

**FIGURE 4:**
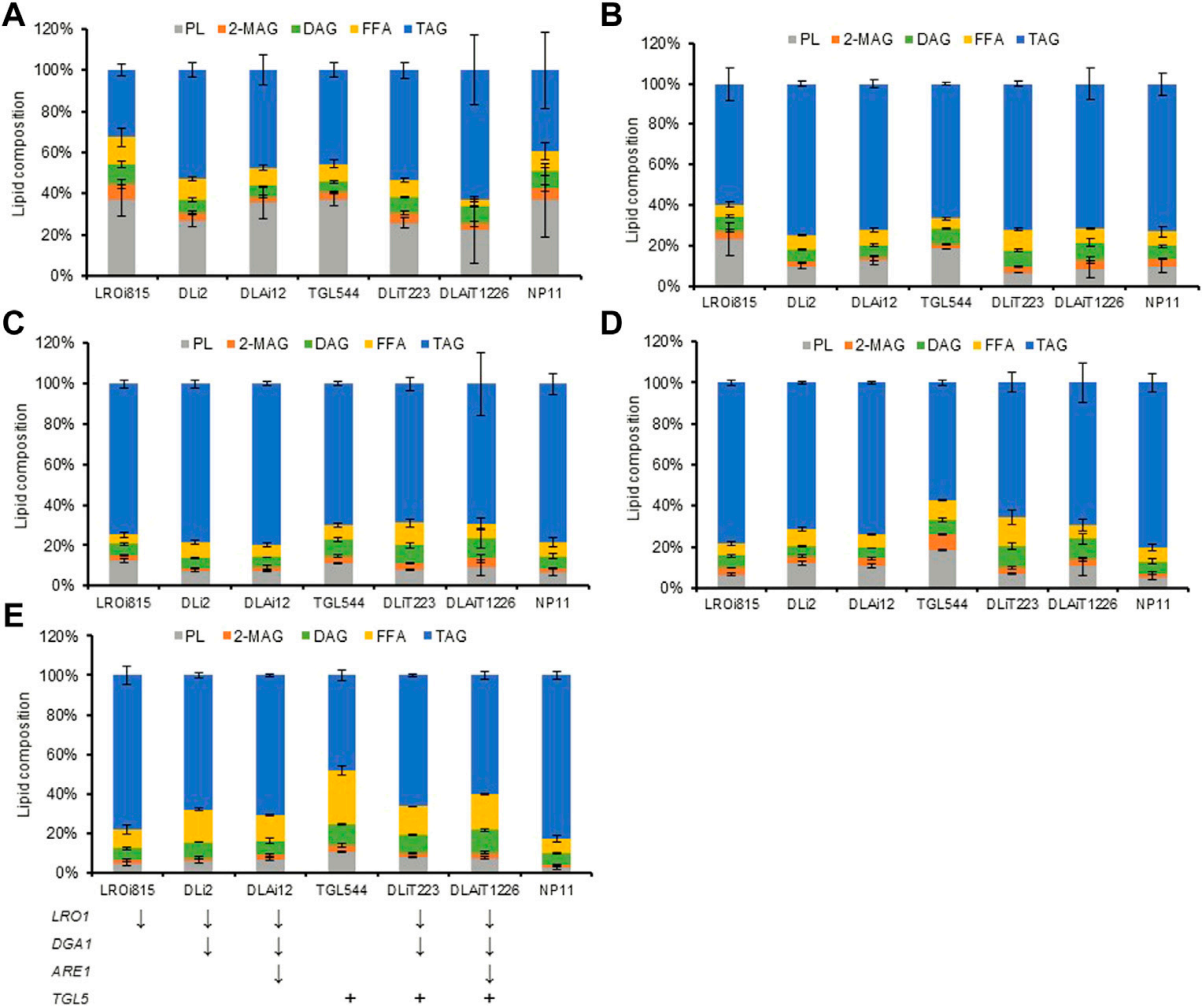
Lipid compositions of strains from 72 h to the end of lipid production under nitrogen limitation. The quenching cells at different stages of lipid production were used for glass beading lipid extraction for analysis of lipid compositions. TAG, triacylglycerols; DAG, diacylglycerols; FFA, free fatty acids; 2-MAG, 2-monoglyceride; PL, polar lipids; **(A)** 72 h **(B)** 96 h **(C)**120 h **(D)** 144 h. **(E)** The ending time.

Compositional profiles of lipids from *R. toruloides* variants changed over time differently (Figure 4). During the lipid accumulation stage from 72 h to 120 h, cellular TAG contents increased rapidly and reached 60% (Figures 4A–C). These differences in lipid compositional profiles might be caused by the rate of lipid accumulation. Meanwhile, the lipid compositional profiles also changed dynamically in engineered strains (Figures 4C–E). The contents of DAG and FFA increased at the late stage of lipid production (after 120 h), especially for those *TGL5* overexpressed strains. In the end, DAG and FFA contents were both improved by up to two-fold in DLAiT1226 cells (Figure 4E). The content (48%) of TAG in strain TGL544 was nearly half of that (82%) of NP11, but the content (27%) of FFA was three-fold more than that (8%) of NP11 (Figure 4E). Moreover, the contents of polar lipids (PL) decreased over time but remained higher than that of NP11 at the late stage. Specifically, PL content (11%) of the strain TGL544 was over three-fold higher than that (3%) of NP11. Intracellular lipid droplets of engineered strains were clearly identified and showed dynamic changes during the lipid accumulation process (Supplementary Figure S3), however, much smaller lipid droplets were noticed in engineered strains than those of NP11. These phenomena were consistent with the expected interconversion amount TAG, DAG, FFA and phospholipids (Figure 2). In addition, fatty acid compositions of strains DLiT223 and DLiT1226 were noticeably different from that of NP11 (Supplementary Table S3). Specifically, considerably lower content of oleic acid (C18:1) but higher contents of very long-chain fatty acids including lignoceric acid (C24:0) were found in lipid products by DLiT223 and DLiT1226. It should be noted that DLiT223 and DLiT1226 were grown much longer time than NP11, such that the fatty acid elongation processes might be actuated for the synthesis of very long chain fatty acids.

The fact that most of the engineered strains showed reduced cell growth and sugar consumption in the lipid production phase indicated that the conversion of glucose into lipids was tightly linked to cell physiology. According to previous studies on the relationship between lipid production and cell growth (Ganger et al., 2002; Zhang et al., 2012), we presumed that the accumulation of TAG might be essential to oleaginous yeasts for lipid production phenotype. Thus, interruption of TAG biosynthetic process could lead to disturbance of lipid production, cell metabolism as well as cell growth. In another aspect, because excess DAG and FFA have been known to interrupt septin assembly, cell division (Yang et al., 2016) and energy metabolism (Schneiter and Kohlwein, 1997), it was reasonable that engineered strains compromised in glucose assimilation, cell growth and TAG accumulation under nitrogen-limited condition.

### The correlation between lipid composition and cell growth

**FIGURE 5:**
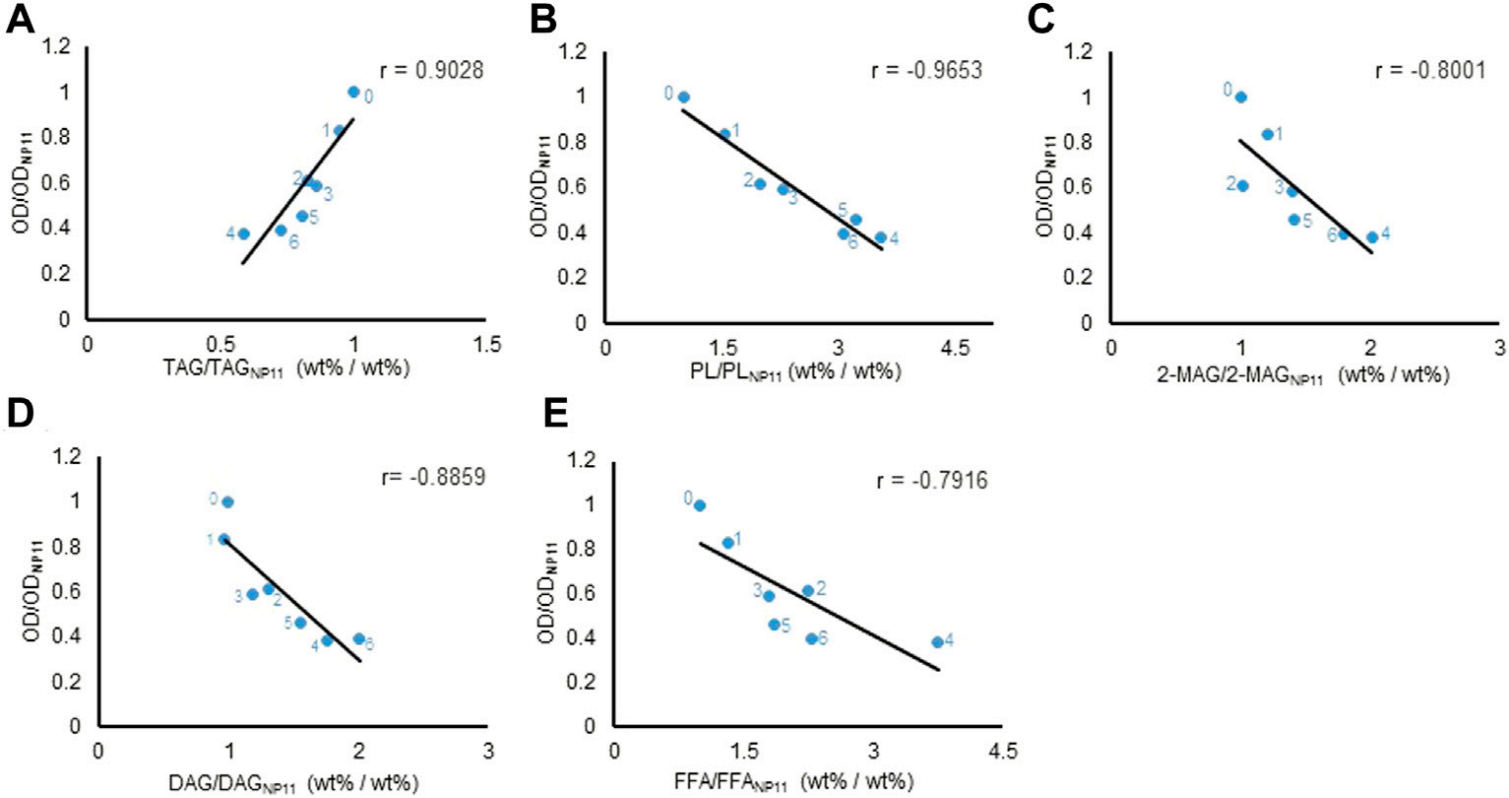
Different types of lipid content correlated with cell growth at the end of lipid production. Pearson correlation analysis between lipids content and OD_600_. Every lipid content and OD_600_ were normalized before Pearson correlation analysis. The horizontal axis was the normalization result of different lipids’ average content of six engineering strains relative to NP11, the vertical axis was the normalization result of OD_600_ average values of six engineering strains relative to NP11. The r value was the Pearson correlation coefficient. The values were plotted on a linear scale graph. **(A)** TAG. **(B)** PL. **(C)** 2-MAG. **(D)** DAG. **(E)** FFA. The number labeled next to the point, 0, 1, 2, 3, 4, 5, and 6 represented strains NP11, LROi815, DLi2, DLAi12, TGL544, DLiT223, and DLAiT1226, respectively. TAG, triacylglycerols; DAG, diacylglycerols; FFA, free fatty acids; 2-MAG, 2-monoglyceride; PL, polar lipids.

To investigate whether there is a quantitative correlation between lipid composition and cell growth, we perform a Pearson correlation analysis of the data from the engineered *R. toruloides* variants. The Pearson product-moment correlation coefficient (r) between TAG contents and cell density at 600 nm (all data were normalized dimension to NP11) at the end of the lipid production culture was 0.9 (Figure 5A), indicating a strong positive correlation between TAG and cell growth. While the contents of others including DAG, FFA, PL and 2-MAG, were all inversely proportional to cell density (Figures 5B–E). When the data of lipid compositional profiles from 72 h to the end of culture were analyzed, they showed similar correlation profiles (Supplementary Figures S5–S8). It was clear that cell growth of these engineered strains correlated positively with TAG content but negatively with the contents of other lipid components, especially DAG. Moreover, when different metrics such as lipid concentration or lipid amount per OD were used for analysis, similar correlation profiles were obtained (Supplementary Figure S9). The information suggested that the accumulation of lipid components other than TAG could have inhibitory effects in *R. toruloides*. In fact, higher cellular contents of lipid species have been known to have side effects on cell metabolism. It has been known that FFA levels need to be maintained carefully to sustain cell activities (Schneiter and Kohlwein., 1997), and increased DAG levels in the budding yeast affected cell growth by intervening septin assembly and cell division (Yang et al., 2016). Similarly, engineered *R. toruloides* strains grown under nitrogen-limited conditions might not divide normally because of the up-regulation of DAG formation.

## Discussion

To explore the capacity of the oleaginous yeast *R. toruloides* for the production of higher-value lipids, we attempted to manipulate its TAG metabolism. In *R. toruloides* genome, three enzymes *DGA1*, *LRO1* and *ARE1*, are found to convert DAG into TAG, while Tgl5p is believed to hydrolyze TAG into DAG and FFA. In this work, we first down-regulated *DGA1* and *LRO1* with one vector through RNAi technology and then down-regulated *ARE1* and overexpressed *TGL5*. We found that engineered strains had little growth defects in nitrogen-rich media but showed reduced growth during the lipid production phase. Compositional analysis indicated that engineered strains produced total lipids in nitrogen-limited media with substantially higher contents of DAG and FFA. These phenomena were consistent with the previous observation (Ganger et al., 2002; Sandager et al., 2002), in which cells failed to store excess fatty acids and DAG or molded abnormal lipid droplets to rescue the cells from FFA toxicity (Connerth et al., 2010; Rani et al., 2013; Yang et al., 2016). Our results suggested that *DGA1* and *LRO1* play similar yet crucial roles in TAG synthesis, but *ARE1* is less important in *R. toruloides*. Moreover, the function of Tgl5p was confirmed to facilitate TAG hydrolysis. Our results also indicated that the formation of TAG correlated strongly positive with cell growth during the lipid production phase, while excess DAG and other lipids, including phospholipids caused cell growth inhibition. Compositions of phospholipids estimated by HPTLC and LC-MS lipidomics method showed little differences in terms of phosphatidylcholine (PC) and phosphatidylethanolamine (PE) contents but higher contents of ceramides (data not shown). These results were different from that obtained in *Rhodotorula glutinis*, where an alteration in TAG biosynthesis led to reduced cell growth but increased contents of PC and PE (Ganger et al., 2002).

It should be noted that the metabolism of DAG and TAG is complex. DAG involves many cellular pathways and dramatically affects cell growth. While we overexpressed Tgl5-Ldp1 for TAG hydrolysis around lipid droplets, it remained unclear whether the products DAG and FFA were stored within lipid droplets or released to the cytoplasm. Meanwhile, the fate and distribution were also elusive for excess DAG formed by knocking down three acyltransferases in endoplasm reticulum. To further regulate DAG production, several strategies may be envisioned. As lipin Pah1p (Adeyo et al., 2011) and DAG kinase Dgk1p (Qiu et al., 2016) are crucial to maintaining cellular DAG and PA levels (Khor et al., 2013; Qiu et al., 2016), manipulation of these two genes can be considered. In addition, it is feasible to delete some TAG biosynthesis-related genes with the CRISPR Cas9 technique developed for *R. toruloides* recently (Jiao et al., 2018). Indeed, blocking TAG biosynthesis has been known to improve the substrate for the production of fatty alcohols (Schultz et al., 2022). In another scenario, when cultivated in phosphorus-limitation media in the previous report (Wu et al., 2010), these engineered strains showed little growth defects and produced lipids with little compositional variation (Supplementary Figure S9). These results were unexpected, although it has been known that the machinery of lipid metabolism in response to nitrogen-limitation and phosphorus-limitation are quite different in *R. toruloides* (Zhu et al., 2012; Wang et al., 2018). Thus, further efforts should be put into more extensive engineering of lipid metabolism as well as a systematic evaluation of cell culture conditions and processes.

Collectively, our work provided clues for modifying the lipid biosynthesis metabolic pathway to improve the production of DAG in *R. toruloides* and enriched our understanding on the correlation between the levels of neutral lipids and cell growth. The information may be applied to restrain TAG production but pursue other value-added products, especially fatty acid-derived chemicals which are more compatible with cell physiology of oleaginous yeasts.

## Data Availability

The original contributions presented in the study are included in the article/Supplementary Material, further inquiries can be directed to the corresponding author.
