# Supplementary material for: Genetic manipulation of the interconversion between diacylglycerols and triacylglycerols in Rhodosporidium toruloides
Source: Front Bioeng Biotechnol. 2022 Oct 26;10:1034972. doi: 10.3389/fbioe.2022.1034972 (PMC9643831; doi:10.3389/fbioe.2022.1034972)
Supplement: Supplementary file 1 [file DataSheet1.pdf]

Supplementary Material

SUPPLEMENTARY TABLES

Supplementary Table S1. The primers used in this study.

Priners	sequences (5'-3')	application
LROi-LRO-p1(EcoRV)	TTCGATATCATGAGCACAGTGCGACGGCGCAACC	Primer used for LRO1-RNAi plasmid construction
LROi-LRO-p2(NcoI)	TTGGCCATGGCGTTGAGAGCGGAAAGGGAAGGCAT	
LROi-inLRO-p1(NcoI)	TTGGCCATGGGGGTCGCCCTCGGTGAACATCCAGC	
LROi-inLRO-p2(SpeI)	TGGACTAGTATGAGCACAGTGCGACGGCGCAACC	
DGAi-DGA-p1(EcoRV)	TTCGATATCATGGGCCAGCAGGCGACGCCCGAGG	Primer used for DGA1-LRO1 RNAi plasmid construction
DGAiLROi-DGA-p2(SOE)	GGTTGCGCCGTCGCACTGTGCTCATGGTGAGGAGGTGAG GGTTCAAGCC	
DGAiLROi-LRO-p1(SOE)	GGCTTGAACCCTCACCTCCTCACCATGAGCACAGTGCGA CGGCGCAACC	
DGAi-inDGA-p2(SpeI)	TGGACTAGTATGGGCCAGCAGGCGACGCCCGAGG	
ARE1-gsen-F	ACACCAGATCAGTCACAGATATCATGTCGACTGTTACCG GCG	Primer used for ARE1-RNAi plasmid construction
ARE1-gsen-R	CACAGGCAAACGGAGAGAACAGCGTCAATCTC	
ARE1-ganti-F	GTTCTCTCCGTTTGCCTGTGACAGTCGTG	
ARE1-ganti-R	ACGGGGCGGAATCGTACTAGTATGTCGACTGTTACCGGC G	
q-DGAi-F	GCGAGAACGACATCTTTGGC	Primer used for quantitative *DGA1* expression
q-DGA-R	GTAGTTGAAGAGTCCCCGGC	
q-LRO-F	CTGGCTTGGATCAGGATGGG	Primer used for quantitative *LRO1* expression
q-LRO-R	CGCAAGTCGCCAATCGTAAG	
qARE1677-F	CTTTGAGGGCGTGTGCAATG	Primer used for quantitative *ARE1* expression
qARE1789-R	GATTCCACTTGCGCGAGAAC	
ACTIN-F	GCTGTCTTCCCCTCGATTGT	Primer used for quantitative *ACTIN* expression
ACTIN-R	GGGTCAGGATACCACGCTTC	
His6-TGL5-F	ACAGATATCATGCACCACCATCACCATCACGAGGCCGAC TTGTCCCGCCCGCCTCCC	Primer used for *TGL5-LDP1* fusion expression plasmid construction
TGL5-R	CGAGGTGAGACGGGGCGGAATCGTACTAGTCTAGTGCCA GGGATCCTCGTCGCTCGA	
TGL5-LDP1-F	GAGGATCCCTGGCACGGCGGCGGCAGCGGCGCCACCGTC AACGAGAAGCA	
LDP1-R	GAGGATCCCTGGCACGGCGGCGGCAGCGGCGCCACCGTC AACGAGAAGCA	
Ppgk-F1	CAAGTGGCTGTAAGCAAGCG	Amplification of *TGL5-LDP1* fusion expression cassette
Ble-R1	GGAACGGCACTGGTCAACTTG	

Table S2. Abbreviations in Figure 2.

Abbreviations		Abbreviations	enzymes encoded genes
GA-3-P	glyceraldehyde 3-phosphate	*GPD1*	glyceraldehyde 3-phosphate dehydrogenase
DHAP	dihydroxyacetonephosphate	*GPD2*	glycerol-3-phosphate dehydrogenase (NAD+)
Ac-CoA	acyl coenzyme A	*GAT1*	glycerol-3-phosphate O-acyltransferase / dihydroxyacetone phosphate acyltransferase
OAA	oxaloacetic acid	*AYR1*	1-acylglycerone phosphate reductase
CIT	citrate	*SLC1*	1-acylglycerol-3-phosphate acyltransferase
ICT	isocitrate	*CDS1*	phosphatidate cytidylyltransferase
α-KG	α-ketoglutarate	*MGAT*	Monoglyceryl transferase
Mal-CoA	malonyl coenzyme A	*LPP1*	lipid phosphatase
Palmitoyl-CoA	palmitoyl coenzyme A	*APP1*	Lysophosphatidylethanolamine Acyltransferase
Acyl-DHAP	acyl dihydroxyacetonephosphate	*DPP1*	diacylglyc-erol pyrophosphate phosphatase
Glycerol-3-P	glycerol 3-phosphate	*ATG15*	triacylglycerol lipase, homolog of ATG15

Table S3. Fatty acid compositions of engineering strains at the end of lipid production. ***p < 0.001, **p < 0.01, *p < 0.05 of one-way factor AVONA.

Fatty acids	NP11	LROi815	DLi2	DLAi12	TGL544	DLiT223	DLAiT1226
C14：0	1.70±0.20	1.69±0.72	1.55±0.36	1.50±0.19	1.11±0.48	0.91±0.04**	0.77±0.45*
C15：0	0.24±0.03	0.28±0.16	0.29±0.06	0.32±0.06	0.38±0.13	0.21±0.01	0.16±0.02*
C16：1	1.19±0.05	1.05±0.20	1.11±0.19	1.11±0.12	1.08±0.49	0.49±0.02***	0.61±0.02***
C16：0	29.95±3.96	30.59±11.70	33.52±6.27	32.96±3.35	27.62±6.97	25.18±0.61	19.05±1.30*
C17：1	0.30±0.03	0.32±0.06	0.45±0.05*	0.51±0.08*	0.44±0.08	0.13±0.01***	0.13±0.03**
C17：0	0.33±0.04	0.41±0.18	0.72±0.09**	0.79±0.11**	0.85±0.10***	0.89±0.07***	0.69±0.09**
C18：2	8.43±6.05	10.03±8.56	3.60±4.54	2.70±2.84	12.92±9.19	15.19±1.25	20.81±0.20*
C18：1	44.52±0.97	41.48±9.93	40.98±5.73	41.51±2.34	34.29±2.08**	26.77±0.64***	27.85±0.93***
C18：0	9.94±1.50	10.29±4.15	12.31±2.38	12.27±1.25	12.43±1.75	20.56±0.64***	16.94±1.36**
C18：3	0.53±0.15	0.21±0.06*	0.38±0.30	0.29±0.09	0.46±0.10	0.37±0.07	0.23±0.03*
C20：0	0.41±0.07	0.57±0.32	0.40±0.32	0.63±0.07*	0.63±0.14	0.90±0.04***	0.87±0.05***
C22：0	0.71±0.08	0.81±0.32	0.82±0.30	1.05±0.10*	1.67±0.18**	2.18±0.12***	2.42±0.38**
C23：0	0.10±0.03	0.14±0.11	0.43±0.42	0.22±0.01**	0.49±0.09**	0.27±0.02**	0.33±0.05**
C24：0	1.37±0.16	1.78±0.69	2.02±1.67	3.36±0.39**	4.43±0.63**	5.00±0.55***	7.36±0.54***
C25：0	0.09±0.00	0.14±0.07	1.10±1.61	0.30±0.03***	0.46±0.09**	0.32±0.06**	0.52±0.08***
C26：0	0.20±0.05	0.22±0.07	0.32±0.12	0.48±0.06**	0.73±0.24*	0.64±0.13**	1.25±0.10***

SUPPLEMENTARY FIGURES

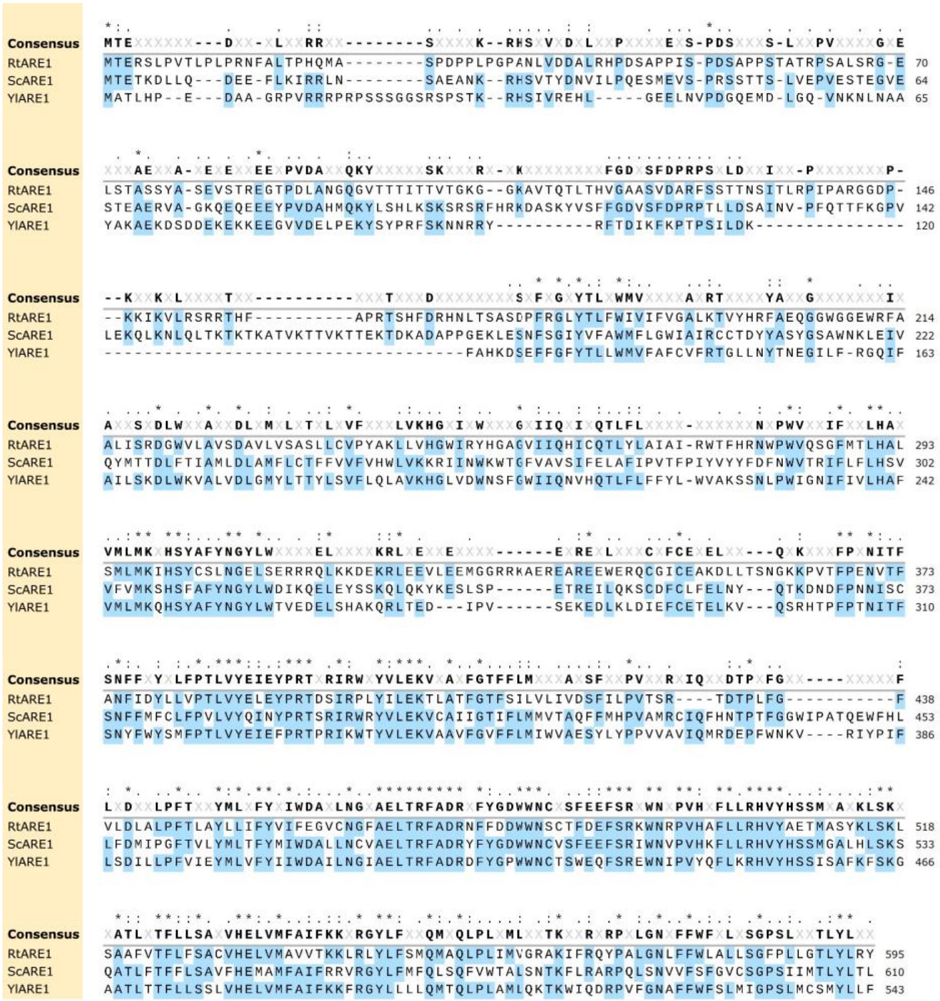

Supplementary Figure S1. Acyl-CoA: sterol acyltransferase Are1 protein amino acid sequence alignment among *Saccharomyces cerevisiae* (Sc), *Yarrowia lipolytica* (Yl), and *R. toruloides* (Rt). The blue highlight marks the consensus amino acids, and the symbols at the top of the amino acids letter showed similarity.

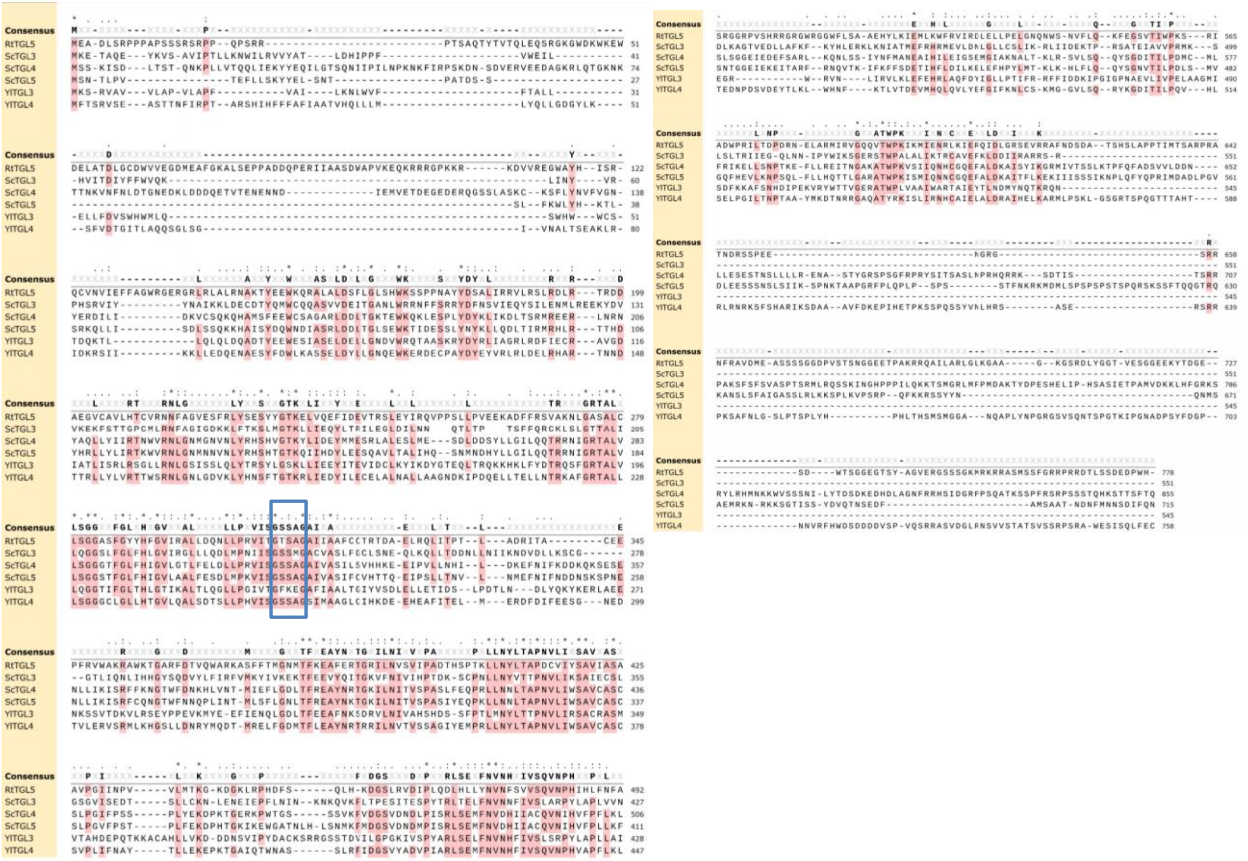

Supplementary Figure S2. TAG lipase (Tgls) protein amino acid sequence alignment among *Saccharomyces cerevisiae* (Sc), *Yarrowia lipolytica* (Yl), and *R. toruloides* (Rt). The red highlight marks the consensus amino acids, and the lipolytic motifs (GXSXG) characteristic of lipolytic enzymes are highlighted with the blue frame.

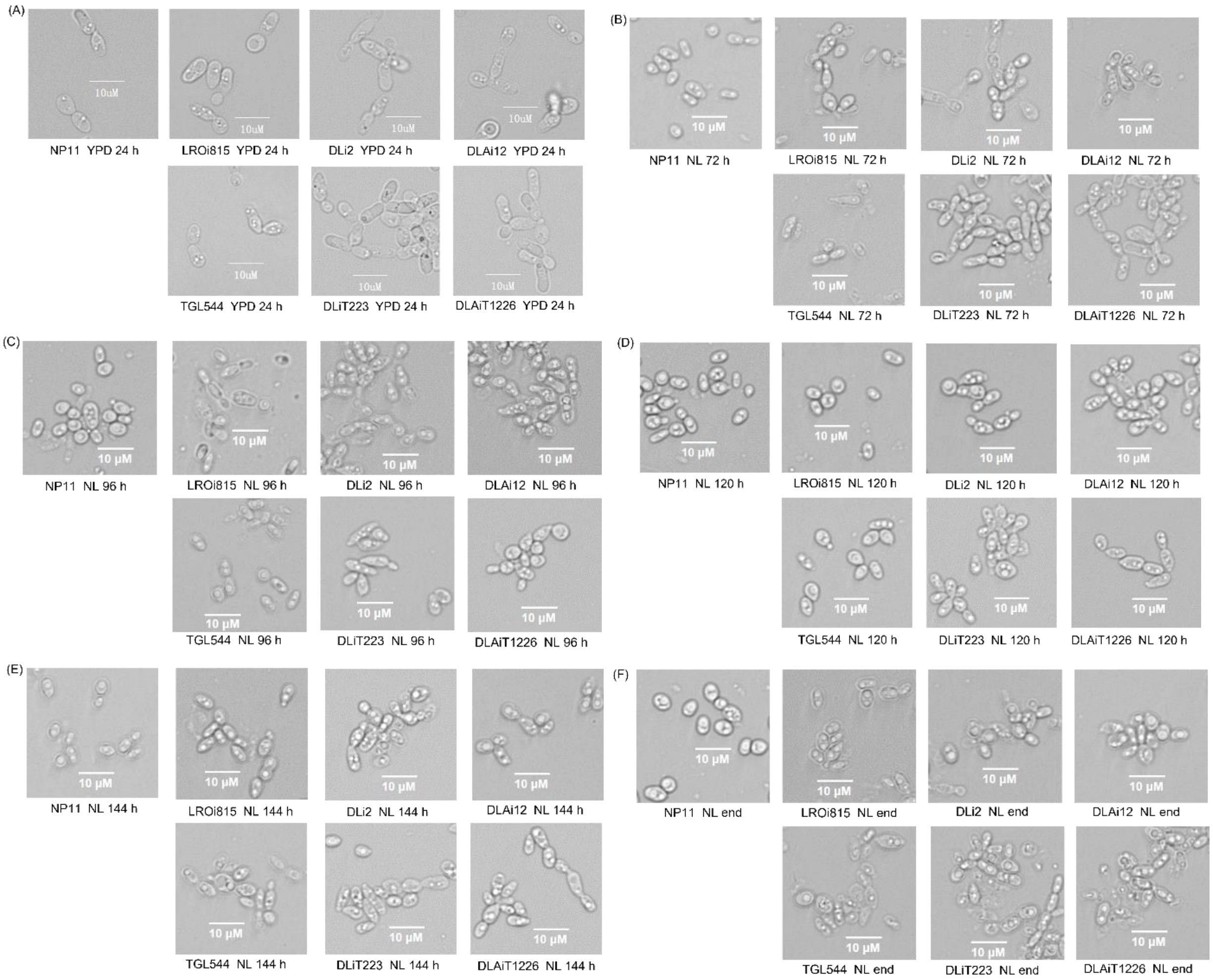

Supplementary Figure S3. Microscope pictures of engineering strains during the lipid production. (A) The engineering strains were cultured in a YPD medium for 24 h as the seed of fermentation. The cellular morphology of strains was observed at different stages of lipid production under nitrogen-limitation (NL) with the microscope. **(B)** 72h. **(C)** 96h. **(D)** 120 h. **(E)** 144h. **(F)** In the end of the culture. The end time of the different strains' cultures was 168 h for NP11 and engineering strains LROi815, 240 h for DLi2 and DLAi12, 288 h for TGL544 and DLiT223, and 360 h for DLAiT1226.

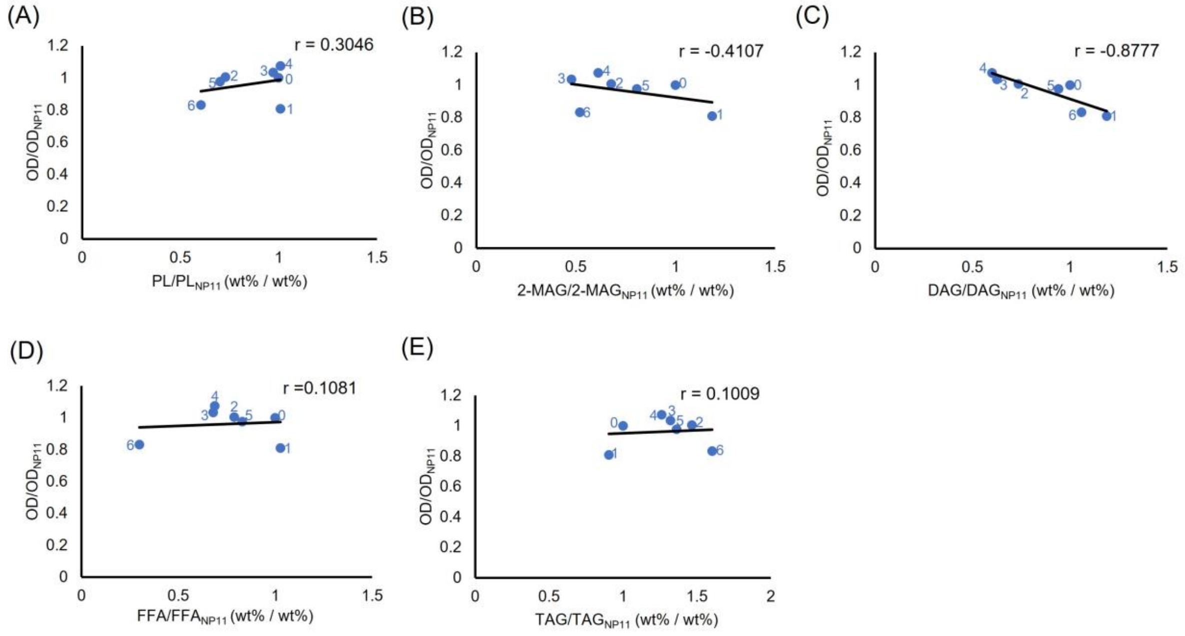

Supplementary Figure S4. Different types of lipids content correlated with cell growth at 72 h of lipid production. Pearson correlation analysis between lipids content and OD_{600} at 72 h of lipid production. Every lipid content and OD_{600} were normalized before Pearson correlation analysis. The horizontal axis was the normalization result of different lipids' average content of engineering strains relative to NP11, the vertical axis was the normalization result of OD_{600} average values of engineering strains relative to NP11. The r value was the Pearson correlation coefficient. The values were plotted on a linear scale graph. **(A)** PL. **(B)** 2-MAG. **(C)** DAG. **(D)** FFA. **(E)** TAG. The number labeled next to the point, 0, 1, 2, 3, 4, 5, and 6 represented strains NP11, LROi815, DLi2, DLAi12, TGL544, DLiT223, and DLAiT1226, respectively. Abbreviation, TAG, triacylglycerols; DAG, diacylglycerols; FFA, free fatty acids; 2-MAG, 2-monoglyceride; PL, polar lipids.

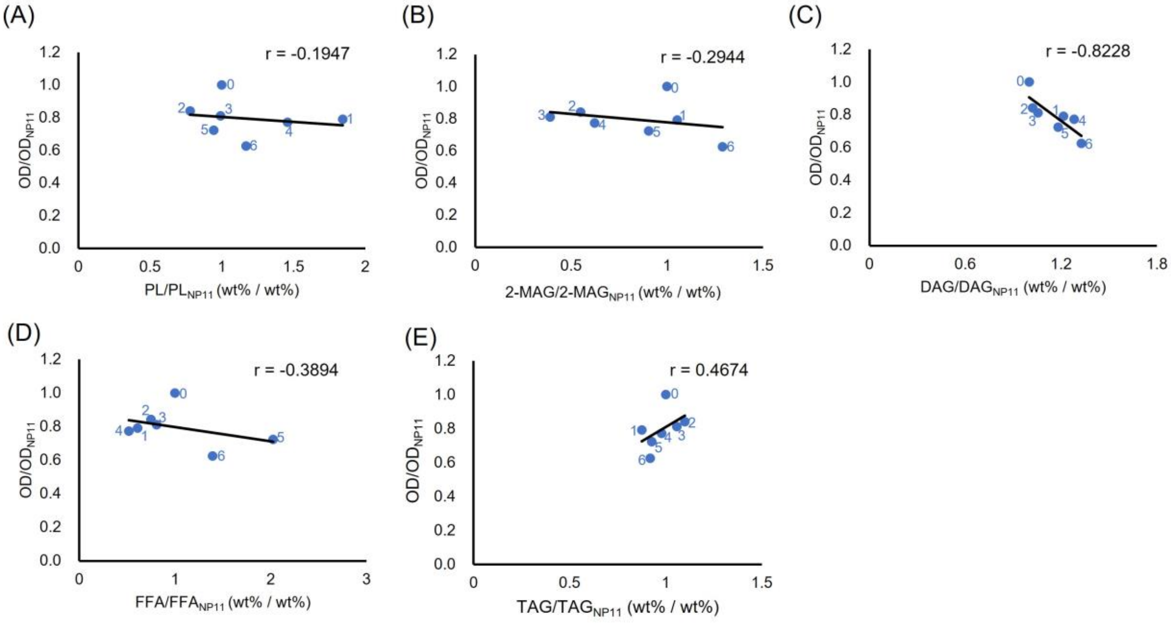

Supplementary Figure S5. Different types of lipids content correlated with cell growth at 96 h of lipid production. Pearson correlation analysis between lipids content and OD_{600} at 96 h of lipid production. Every lipid content and OD_{600} were normalized before Pearson correlation analysis. The horizontal axis was the normalization result of different lipids' average content of engineering strains relative to NP11, the vertical axis was the normalization result of OD_{600} average values of engineering strains relative to NP11. The r value was the Pearson correlation coefficient. The values were plotted on a linear scale graph. **(A)** PL. **(B)** 2-MAG. **(C)** DAG. **(D)** FFA. **(E)** TAG. The number labeled next to the point, 0, 1, 2, 3, 4, 5, and 6 represented strains NP11, LROi815, DLi2, DLAi12, TGL544, DLiT223, and DLAiT1226, respectively. Abbreviation, TAG, triacylglycerols; DAG, diacylglycerols; FFA, free fatty acids; 2-MAG, 2-monoglyceride; PL, polar lipids.

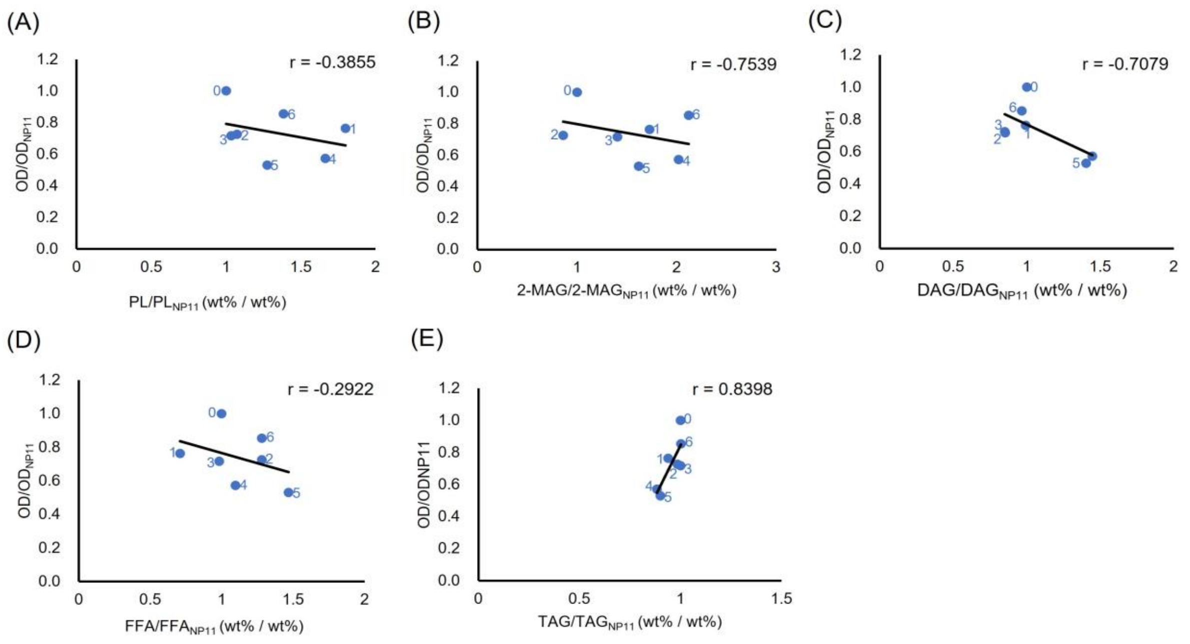

Supplementary Figure S6. Different types of lipids content correlated with cell growth at 120 h of lipid production. Pearson correlation analysis between lipids content and OD_{600} at 120 h of lipid production. Every lipid content and OD_{600} were normalized before Pearson correlation analysis. The horizontal axis was the normalization result of different lipids' average content of engineering strains relative to NP11, the vertical axis was the normalization result of OD_{600} average values of engineering strains relative to NP11. The r value was the Pearson correlation coefficient. The values were plotted on a linear scale graph. **(A)** PL. **(B)** 2-MAG. **(C)** DAG. **(D)** FFA. **(E)** TAG. The number labeled next to the point, 0, 1, 2, 3, 4, 5, and 6 represented strains NP11, LROi815, DLi2, DLAi12, TGL544, DLiT223, and DLAiT1226, respectively. Abbreviation, TAG, triacylglycerols; DAG, diacylglycerols; FFA, free fatty acids; 2-MAG, 2-monoglyceride; PL, polar lipids.

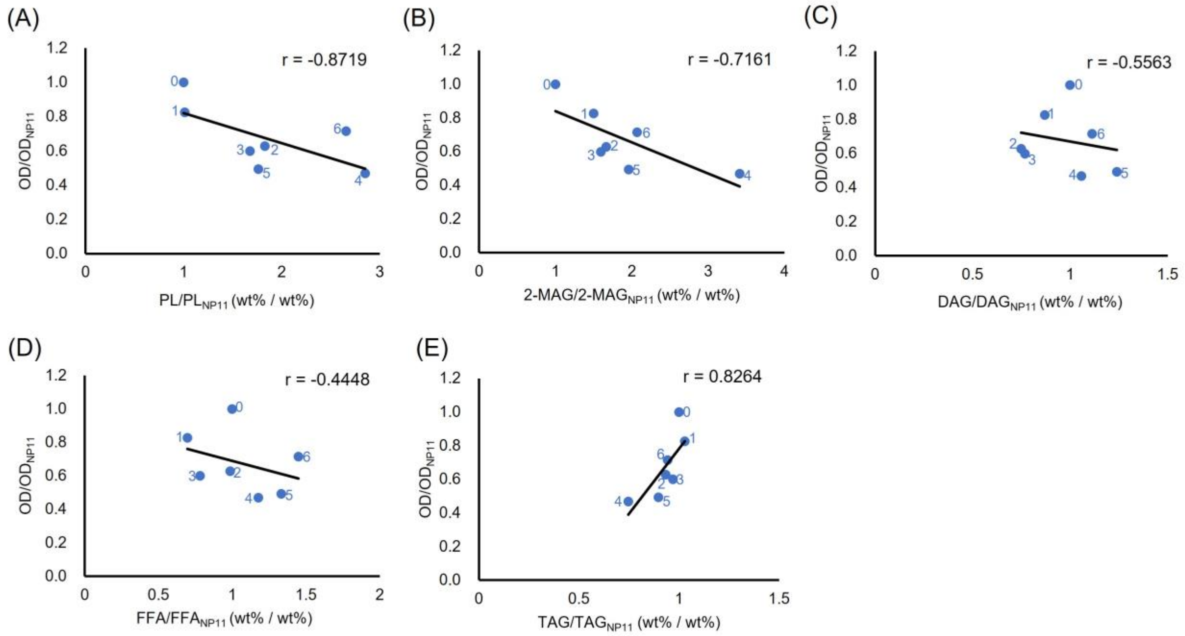

Supplementary Figure S7. Different types of lipids content correlated with cell growth at 144 h of lipid production. Pearson correlation analysis between lipids content and OD_{600} at 144 h of lipid production. Every lipid content and OD_{600} were normalized before Pearson correlation analysis. The horizontal axis was the normalization result of different lipids' average content of engineering strains relative to NP11, the vertical axis was the normalization result of OD_{600} average values of engineering strains relative to NP11. The r value was the Pearson correlation coefficient. The values were plotted on a linear scale graph. **(A)** PL. **(B)** 2-MAG. **(C)** DAG. **(D)** FFA. **(E)** TAG. The number labeled next to the point, 0, 1, 2, 3, 4, 5, and 6 represented strains NP11, LROi815, DLi2, DLAi12, TGL544, DLiT223, and DLAiT1226, respectively. Abbreviation, TAG, triacylglycerols; DAG, diacylglycerols; FFA, free fatty acids; 2-MAG, 2-monoglyceride; PL, polar lipids.

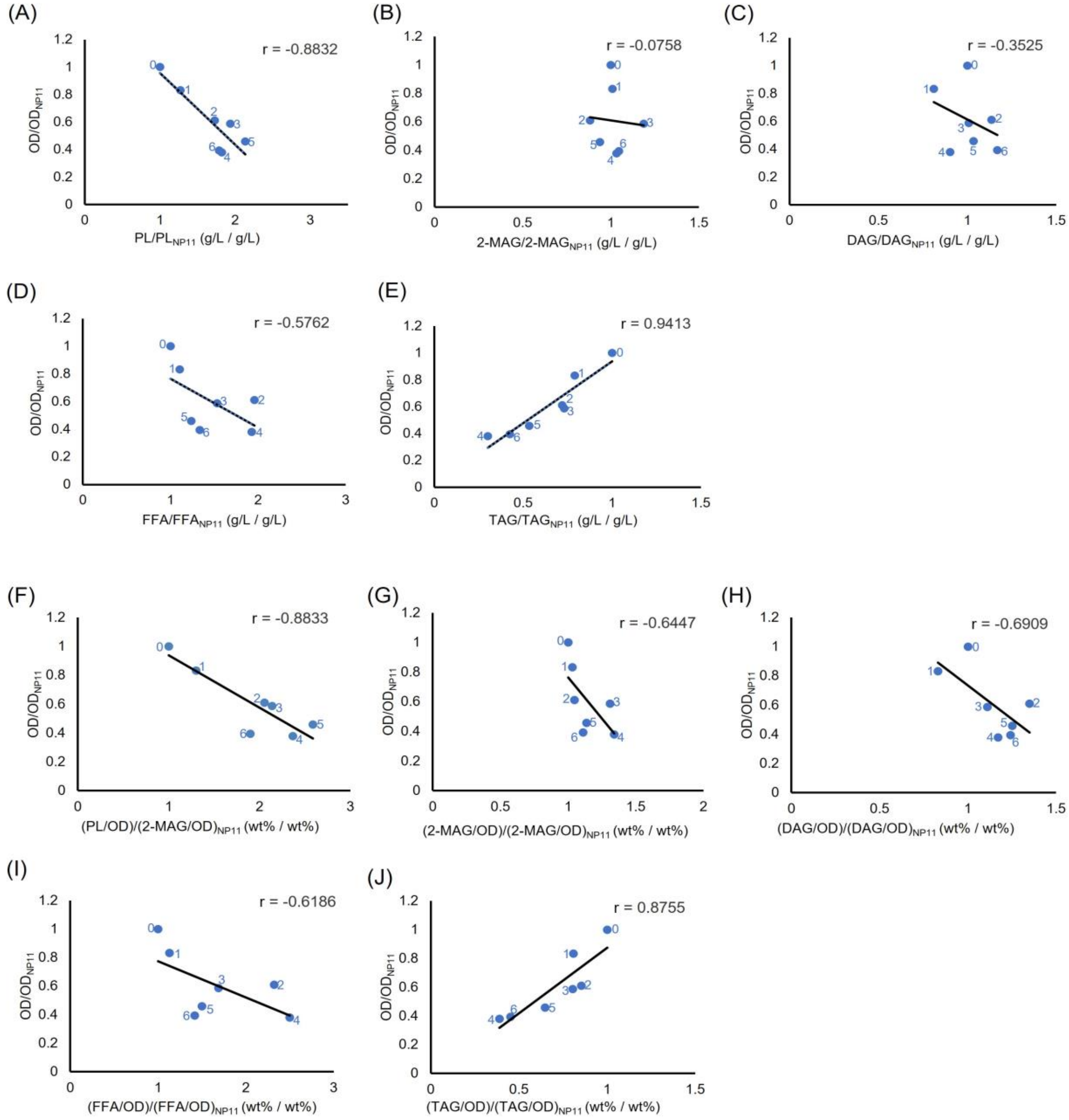

Supplementary Figure S8. Pearson correlation analysis of lipid concentration or lipid content per-OD with OD_{600} at the end of lipid production. (A-E) Relationship between lipid concentration and OD_{600} at the end of lipid production. The horizontal axis was the normalization result of different lipids' average concentration of engineering strains relative to NP11, the vertical axis was the normalization result of OD_{600} average values of engineering strains relative to NP11. The r value was the Pearson correlation coefficient. **(A)** PL. **(B)** 2-MAG. **(C)** DAGs. **(D)** FFA. **(E)** TAG. **(F-J)** Relationship between lipid content per-OD and OD_{600}. The horizontal axis was the normalization result of different lipids' average content of engineering strains relative to NP11, the vertical axis was the same as above. **(F)** PL. **(G)** 2-MAG. **(H)** DAGs. **(I)** FFA. **(J)** TAG The number labeled next to the point, 0, 1, 2, 3, 4, 5, and 6 represented strains NP11, LROi815, DLi2, DLAi12, TGL544, DLiT223, and DLAiT1226, respectively. Abbreviation, TAG, triacylglycerols; DAG, diacylglycerols; FFA, free fatty acids; 2-MAG, 2-monoglyceride; PL, polar lipids.

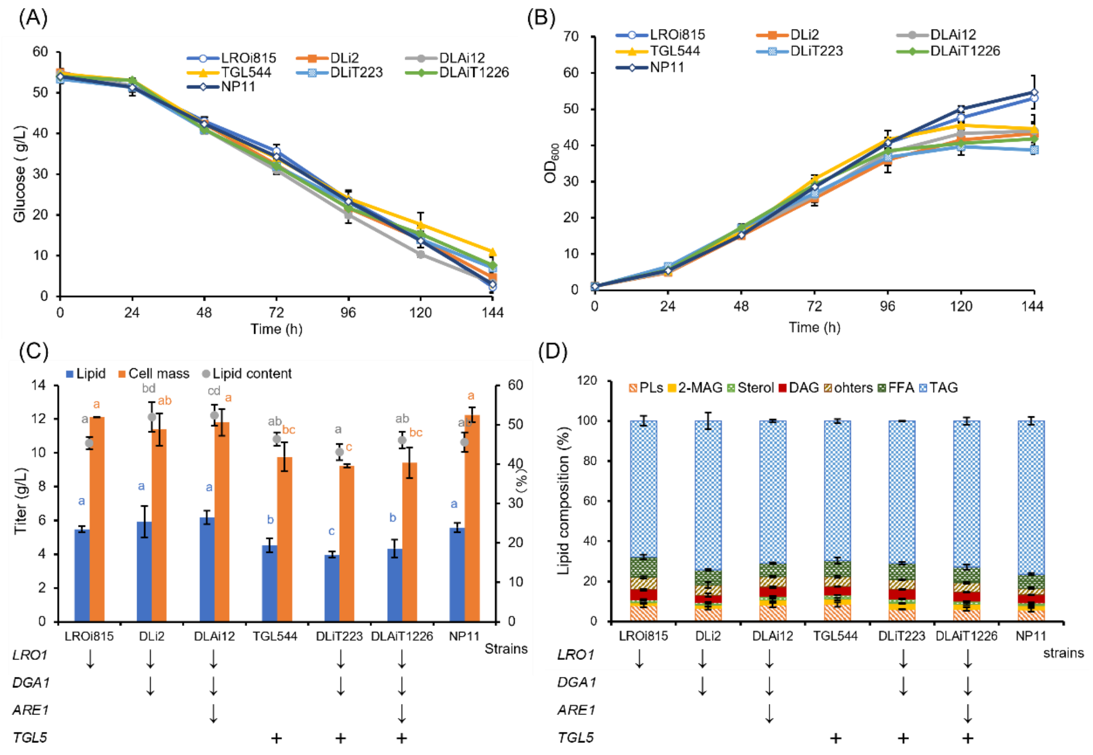

Supplementary Figure S9. The lipid production of engineered strains under the Pi-limited condition with shaking flask cultures. (A) Residual glucose curve in phosphorus-limitation (PL) media, the glucose concentration of supernatant of fermentation broth was detected every 24 h. **(B)** The growth curve of strains under Pi-limitation, growth was measured as the turbidity at 600 nm every 24 h. **(C)** Cell mass, lipid titer, and total lipid content of stains in PL culture. Cell mass was dry cell mass weight of 30 mL fermentation broth, lipid titer was calculated with lipid extracted from the above dry cell by acid heating. **(D)** Lipid compositional profiles of stains in PL culture. Means without a common superscript letter and with * differ significantly ($p < 0.05$) as analyzed by the one-way factor AVONA. The gene with ↓ was knocked down, and + was overexpressed.

DNA sequences of LRO1i, DGA1i-LRO1i, ARE1i, and TGL5-LDP1 expression cassettes (from LB to RB) as shown in Figure 2A.

(1) LRO1i expression cassette sequence:

TGGCTGGTGGCAGGATATATTGTGGTGTAAACAAATTGACGCTTAGACAACTTAATAACACATTGCGGACGTTTTTAATGT
ACTGAATTAACGCCGAATTGAATTCGAGCTCGGTACCCGGGCCAGACGGACCTTGAGAACCCTCAATCGCTCGCGGTACTC
GTCCGCCCTGCGATCCAGCATCGAAACCGAGTGCAGCGCGTTCAACAAATCCGAGTCGTCTCCTCCTGCTCCTTCGCGCTG
TTTCGGCGCGGGTGGCGCAGGGACAGCCGGAGGGAGAGGGGGAGGAGGAGGAGGGGCTGGGGCGACCTTTTTCTTCTTCTT
CCTGTTCTTGCCCTTCTTCTTCGCCGCCTCTGCTTCTCCATCACCCACCGCCCCCACATTCGCCGCCGAAGCACCGACCAC
GGCCCCCCTCATCCGCCTCTGCAACCTCCTCGCCTCGCTCGCCTCCAAACTCAATCGCGCGACGCACTGCTCCAACTCGGC
GATGGCGCTCATCAAGCTTGGGAGGGAGGCGGGTGAGAGAGCCGAGTCGGAGAGGATGCCGTCTGCGGGGATTTGGGAGGG
TGAGAGGTGGGTTTGAGGCGGTTGAGAGGAGGAGGACGAGAGGGGGAGGGCGGAGGAGAGGGCTGTCAAGTCCGAGAGGGA
GAGGGGGATCGAGGTCGTGAAGGATGTCGAGGACGAGGAAGAGGCGTTCCGCTGTTGTTGTTGCTGGACGGCGGAGAGGAC
GCCCAGGAAGGCTGCGTCGGCCGCTTGCGAGGGCGAGTCGATCACCTCGTAGGCGGCGTCGTCGTGCTCGGACGGCGACGA
CGCGTGGTCCTCCGACTCGACCCAGTCTGGCCCGTTGAAGAGGCATTCGGCGGCGAAGACGACGGCCGGGTCGTCCGAGGC
TGTCGAGGCAAGTGGCTGTAAGCAAGCGCGGCAGAAGAGGCAGAACGCTGCGACGCACTCCTAGGATCCCACGGCTGCGCA
TCCGGATCCTCCCCGCGCTGCTGGCGCCTCGCCCGCTCCCGCTCGTACTCCGCCATGACCGTCAACCCCTGGCAGAGCAGG
CGGTACTGCTCGACCAGGGCCCAGTCCTCGCTCGTCTGTGGGAAAGGTTGGACGGGACATCCTGGCGGCAATGGAAGCGAG
TACAGCCAGTCGAGCGTGAGAGGGGAGGGCCGAAAGTGCTCCGTCGAGATGCTGGCGAGCGAGACGAGGGGCTGGGTGAGC
GACGGCGGGCGGATCCCTGCGCCTGTGTTGTCCATCCCTGCAGTGCACTCTGTTGCTCGTATCATGTCCCACTCCCTTGTA
TCCCTCGAGTCGGTCGACTCTTCCCTGGCGAGTCCAAGCGGAGGAGGTGGTCGTCGCCTGACCCGCTCGGAGTGCGCCGCT
CGACTTGGCCCTGGGAGAACAAGCCTGTGTGAGTCTGTCTAGCCTGTCAGCGAATGCGCCAGACGAGTGCAAGCGGGTGAG
CGAGGTCGACCCTGCTCGTCACTCGCTCGTCGGGTGCGGCCGCATCGTTGAACTTGCACTTCTCACTCGCACTCGCTCTGG
TACAGCTACAGTCACTCGCTTACTACTCTGCAGGTTCACAGCAACTCACCCGTCCAACTCCCACCCTCCCCCGTGCAGCCC
ACCATGCCGGAGCTCACGGCGACGTCGGTCGAGAAGTTCCTCATCGAAAAGTTCGACTCGGTCTCGGACCTCATGCAACTC
TCGGAGGGAGAGGAATCGCGCGCGTTCTCGTTCGACGTCGGAGGCCGCGGATACGTCCTCCGCGTCAACTCGTGTGCGGAC
GGATTCTACAAGGATCGGTACGTCTACCGCCATTTTGCGTCGGCGGCGCTCCCGATCCCCGAAGTCCTCGACATCGGAGAG
TTCTCGGAATCCCTCACGTACTGTATCTCGCGCCGGGCGCAAGGAGTCACGCTCCAAGATCTCCCGGAGACGGAACTCCCG
GCGGTCCTCCAACCGGTCGCGGAAGCGATGGACGCGATCGCGGCCGCGGACCTCTCGCAAACGTCGGGATTCGGACCGTTT
GGACCGCAAGGAATCGGACAATACACGACGTGGCGCGACTTCATCTGTGCGATCGCGGATCCCCATGTCTACCACTGGCAA
ACGGTCATGGATGACACGGTCTCGGCGTCGGTCGCGCAAGCGCTCGACGAGCTCATGCTCTGGGCGGAGGACTGTCCGGAG
GTCCGCCACCTCGTCCACGCGGACTTTGGATCGAACAACGTCCTCACGGACAACGGACGCATCACGGCGGTCATCGACTGG
TCGGAGGCGATGTTTGGAGACTCGCAATACGAGGTCGCGAACATCTTCTTCTGGCGCCCGTGGCTCGCGTGCATGGAGCAA
CAGACGCGCTACTTCGAGCGCCGCCACCCGGAGCTCGCGGATCGCCGCGCCTCCGCGCGTACATGCTCCGCATCGGCCTC
GACCAACTCTACCAGTCGCTCGTCGACGGAAACTTCGACGATGCCGCGTGGGCCCAAGGACGCTGCGACGCGATTGTCCGC
TCGGGAGCGGGAACCGTGGGACGCACGCAAATTGCGCGGCGCTCGGCGGCCGTCTGGACGGATGGATGTGTCGAAGTCCTC
GCGGATTCGGGAAACCGGCGCCCGTCGACGCGCCCGCGGGCGAAAGAACACCACCATCACCATCACTAGGATCGTTCAAAC
ATTTGGCAATAAAGTTTCTTAAGATTGAATCCTGTTGCCGGTCTTGCGATGATTATCATATAATTTCTGTTGAATTACGTT
AAGCATGTAATAATTAACATGTAATGCATGACGTTATTTATGAGATGGGTTTTTATGATTAGAGTCCCGCAATTATACATT
TAATACGCGATAGAAAACAAAATATAGCGCGCAAACTAGGATAAATTATCGCGCGCGGTGTCATCTATGTTACTAGATCGG
GCCTGGATCCTCTAGATCCATGCTGCTGCGATCTGGGAGTGCAAGCCCGCGGTCAGCAGCCATTGCCCGCCTACAATGTCT
TCCCAAAGCCGCCCTTGCTCGCTCGCTGCGTGGAGTTCGACGTTCTCCTCGCTCCGCAAGCATTGGAATGAACCTTGCTCT
CTAGTTCCCTCCTCCGTGACCTCGTTTCGTCCTTTAGACGGCACGATGGAAGGAAGAAATCTCTGCGGACAAGCAAATCTG
CTGGCTCGCCTTGTAGGTCGCCTACCGGAGCAAGCCTTGTGCCGCCGGGATGCCAACGTCGTTTTTTGACGTTTGCAAGAC
GTAGAGGACGCTTCGGACGACGAAACAAGCTGTGAGGACATGGAAGTCGTGGGAGGAACGGCGCAGAGCGGCGCCGCGGGA
GCATAAGGCAAGCGAGATAGTCCAGAAATCGCGGCGCCAAGTACAGTAATTTATTGGAGCAGGCACCAGAAGCGGGCAGCA
GTATGCGCAGGCTTGGGGTCGACGAGAGACGACTCCCTCATACTCGGTTACCTCGAGCAATACAATCAATCGAAGCTGCGC
GAATCTCGGCTTGTAAGGGTCGGAAAGGAACCTCGGAGATGGCCACGTCACATCACCAACTTATCGATCTCAGCCGACGTC
GCAGAGAGGGCGAGCGAAGCGGTGAAGGAGGGAAACAATCCCTCGAGAGCATGATCCGTCTGAATCTGCAGCGCAGGAAGC
CGTCACACGCCCGCCTCGAGCGCAGGTCGGGTCCAGCCGGGGGACGAAACGCGCGAGGGCTGATTTCGTGAGCGAAGGAAG
CCGCATCGACAAGTTCGCGTCCCTTTGCCCTCTTTCCCATCACCCGCTCTCGCTCTACCCGCTCAGAACAACACCAGATCA
GTCACAGATATCATGAGCACAGTGCGACGGCGCAACCAGCCAAAGCGCAACGCCTCAGCGCCCGCCAACCAGCTCGATACC
GACGATTCCTCGCCCGCTCCCTCTCGACCTGACTCGCCCGATGTTGACAAAGCGCCAGTGAGGGAGGTCCCGCTGTTCAAG
CGGAGGCTGAAGAAGGGGGATGGCAAGGTGGAAGACCCGAAGGTCATTCTGGACGAGAAGACGGGCAGGGTTTCTCTCGAC
TTTCCAAGGACGCCCTACGACCAGCTCGACCTGAGCGATTCGTTCCTCGCCGCCTTGCACGAGGCACCCTTCGAGCCGAAG
AAGACGTGGACAAAGCGGCGGAGAGGCTGGTTCTTCTTGGGAGGATTGCTCGGTCTCTGCGCAGGCTGGATGTTCACCGAG
GGCGACCCACTCGCCTCTCTCGCCAATCTCGACCTCGATGCCTTCTCATCCTGGGATCTTCAGTCAATCCTCGCCGACATG

CCTTCCCTTTCCGCTCTCAACGCCATGGGGGTCGCCCTCGGTGAACATCCAGCCTGCGCAGAGACCGAGCAATCCTCCCAA
GAAGAACCAGCCTCTCCGCCGCTTTGTCCACGTCTTCTTCGGCTCGAAGGGTGCCTCGTGCAAGGCGGCGAGGAACGAATC
GCTCAGGTCGAGCTGGTCGTAGGGCGTCCTTGGAAAGTCGAGAGAAACCCTGCCCGTCTTCTCGTCCAGAATGACCTTCGG
GTCTTCCACCTTGCCATCCCCCTTCTTCAGCCTCCGCTTGAACAGCGGGACCTCCCTCACTGGCGCTTTGTCAACATCGGG
CGAGTCAGGTCGAGAGGGAGCGGGCGAGGAATCGTCGGTATCGAGCTGGTTGGCGGGCGCACTAGTACGATTCCGCCCCGT
CTCACCTCGCATCCGACCTGTGTAGCCACGCCTTTTCTCTTTCCGCCCCCGACACTAAAAGGAGTTCAGTCGTCCGCTTTT
CCTCTCTTCCGTTCTCCACGCCTGCAGTACTGCATGCCTCAGCTGTGTAATTTCGATAGAGTACTCGCACGTATGCTCGCG
CGGACTTGTCGAGAGAGCGGGCGAGAGGTCGAGAGGAGCTGACTTGAGTGAGCAGCGAGAGCGGAGCGGCTTGTTGCGGCA
CACAACGGTCCGGCTTACGGACAATCAGCGATGAAGCCGGAAGAGCGGCAGGAGTGGACGAAGACGTGCGAAGAGGAAAGG
GGGCGAGGAAGAGAAGCGGAGGAGGAAACGCAGCGAAGCAGCACAACTTCCGCAGAGACGACGGTTCACATAGTCAAAGAT
GCAGTGCAGAGAAGTGCGCGCCGCCTTCAGTTTAAACTATCAGTGTTTGACAGGATATATTGGCGGGTAAAC

(2) DGA1i-LRO1i expression cassette sequence:

TGGCTGGTGGCAGGATATATTGTGGTGTAAACAAATTGACGCTTAGACAACTTAATAACACATTGCGGACGTTTTTAATGT
ACTGAATTAACGCCGAATTGAATTCGAGCTCGGTACCCGGGCCAGACGGACCTTGAGAACCCTCAATCGCTCGCGGTACTC
GTCCGCCCTGCGATCCAGCATCGAAACCGAGTGCAGCGCGTTCAACAAATCCGAGTCGTCTCCTCCTGCTCCTTCGCGCTG
TTTCGGCGCGGGTGGCGCAGGGACAGCCGGAGGGAGAGGGGGAGGAGGAGGAGGGGCTGGGGCGACCTTTTTCTTCTTCTT
CCTGTTCTTGCCCTTCTTCTTCGCCGCCTCTGCTTCTCCATCACCCACCGCCCCCACATTCGCCGCCGAAGCACCGACCAC
GGCCCCCCTCATCCGCCTCTGCAACCTCCTCGCCTCGCTCGCCTCCAAACTCAATCGCGCGACGCACTGCTCCAACTCGGC
GATGGCGCTCATCAAGCTTGGGAGGGAGGCGGGTGAGAGAGCCGAGTCGGAGAGGATGCCGTCTGCGGGGATTTGGGAGGG
TGAGAGGTGGGTTTGAGGCGGTTGAGAGGAGGAGGACGAGAGGGGGAGGGCGGAGGAGAGGGCTGTCAAGTCCGAGAGGGA
GAGGGGGATCGAGGTCGTGAAGGATGTCGAGGACGAGGAAGAGGCGTTCCGCTGTTGTTGTTGCTGGACGGCGGAGAGGAC
GCCCAGGAAGGCTGCGTCGGCCGCTTGCGAGGGCGAGTCGATCACCTCGTAGGCGGCGTCGTCGTGCTCGGACGGCGACGA
CGCGTGGTCCTCCGACTCGACCCAGTCTGGCCCGTTGAAGAGGCATTCGGCGGCGAAGACGACGGCCGGGTCGTCCGAGGC
TGTCGAGGCAAGTGGCTGTAAGCAAGCGCGGCAGAAGAGGCAGAACGCTGCGACGCACTCCTAGGATCCCACGGCTGCGCA
TCCGGATCCTCCCCGCGCTGCTGGCGCCTCGCCCGCTCCCGCTCGTACTCCGCCATGACCGTCAACCCCTGGCAGAGCAGG
CGGTACTGCTCGACCAGGGCCCAGTCCTCGCTCGTCTGTGGGAAAGGTTGGACGGGACATCCTGGCGGCAATGGAAGCGAG
TACAGCCAGTCGAGCGTGAGAGGGGAGGGCCGAAAGTGCTCCGTCGAGATGCTGGCGAGCGAGACGAGGGGCTGGGTGAGC
GACGGCGGGCGGATCCCTGCGCCTGTGTTGTCCATCCCTGCAGTGCACTCTGTTGCTCGTATCATGTCCCACTCCCTTGTA
TCCCTCGAGTCGGTCGACTCTTCCCTGGCGAGTCCAAGCGGAGGAGGTGGTCGTCGCCTGACCCGCTCGGAGTGCGCCGCT
CGACTTGGCCCTGGGAGAACAAGCCTGTGTGAGTCTGTCTAGCCTGTCAGCGAATGCGCCAGACGAGTGCAAGCGGGTGAG
CGAGGTCGACCCTGCTCGTCACTCGCTCGTCGGGTGCGGCCGCATCGTTGAACTTGCACTTCTCACTCGCACTCGCTCTGG
TACAGCTACAGTCACTCGCTTACTACTCTGCAGGTTCACAGCAACTCACCCGTCCAACTCCCACCCTCCCCCGTGCAGCCC
ACCATGCCGGAGCTCACGGCGACGTCGGTCGAGAAGTTCCTCATCGAAAAGTTCGACTCGGTCTCGGACCTCATGCAACTC
TCGGAGGGAGAGGAATCGCGCGCGTTCTCGTTCGACGTCGGAGGCCGCGGATACGTCCTCCGCGTCAACTCGTGTGCGGAC
GGATTCTACAAGGATCGGTACGTCTACCGCCATTTTGCGTCGGCGGCGCTCCCGATCCCCGAAGTCCTCGACATCGGAGAG
TTCTCGGAATCCCTCACGTACTGTATCTCGCGCCGGGCGCAAGGAGTCACGCTCCAAGATCTCCCGGAGACGGAACTCCCG
GCGGTCCTCCAACCGGTCGCGGAAGCGATGGACGCGATCGCGGCCGCGGACCTCTCGCAAACGTCGGGATTCGGACCGTTT
GGACCGCAAGGAATCGGACAATACACGACGTGGCGCGACTTCATCTGTGCGATCGCGGATCCCCATGTCTACCACTGGCAA
ACGGTCATGGATGACACGGTCTCGGCGTCGGTCGCGCAAGCGCTCGACGAGCTCATGCTCTGGGCGGAGGACTGTCCGGAG
GTCCGCCACCTCGTCCACGCGGACTTTGGATCGAACAACGTCCTCACGGACAACGGACGCATCACGGCGGTCATCGACTGG
TCGGAGGCGATGTTTGGAGACTCGCAATACGAGGTCGCGAACATCTTCTTCTGGCGCCCGTGGCTCGCGTGCATGGAGCAA
CAGACGCGCTACTTCGAGCGCCGCCACCCGGAGCTCGCGGGATCGCCGCGCCTCCGCGCGTACATGCTCCGCATCGGCCTC
GACCAACTCTACCAGTCGCTCGTCGACGGAAACTTCGACGATGCCGCGTGGGCCCAAGGACGCTGCGACGCGATTGTCCGC
TCGGGAGCGGGAACCGTGGGACGCACGCAAATTGCGCGGCGCTCGGCGGCCGTCTGGACGGATGGATGTGTCGAAGTCCTC
GCGGATTCGGGAAACCGGCGCCCGTCGACGCGCCCGCGGGCGAAAGAACACCACCATCACCATCACTAGGATCGTTCAAAC
ATTTGGCAATAAAGTTTCTTAAGATTGAATCCTGTTGCCGGTCTTGCGATGATTATCATATAATTTCTGTTGAATTACGTT
AAGCATGTAATAATTAACATGTAATGCATGACGTTATTTATGAGATGGGTTTTTATGATTAGAGTCCCGCAATTATACATT
TAATACGCGATAGAAAACAAAATATAGCGCGCAAACTAGGATAAATTATCGCGCGCGGTGTCATCTATGTTACTAGATCGG
GCCTGGATCCTCTAGATCCATGCTGCTGCGATCTGGGAGTGCAAGCCCGCGGTCAGCAGCCATTGCCCGCCTACAATGTCT
TCCCAAAGCCGCCCTTGCTCGCTCGCTGCGTGGAGTTCGACGTTCTCCTCGCTCCGCAAGCATTGGAATGAACCTTGCTCT
CTAGTTCCCTCCTCCGTGACCTCGTTTCGTCCTTTAGACGGCACGATGGAAGGAAGAAATCTCTGCGGACAAGCAAATCTG
CTGGCTCGCCTTGTAGGTCGCCTACCGGAGCAAGCCTTGTGCCGCCGGGATGCCAACGTCGTTTTTTGACGTTTGCAAGAC
GTAGAGGACGCTTCGGACGACGAAACAAGCTGTGAGGACATGGAAGTCGTGGGAGGAACGGCGCAGAGCGGCGCCGCGGGA
GCATAAGGCAAGCGAGATAGTCCAGAAATCGCGGCGCCAAGTACAGTAATTTATTGGAGCAGGCACCAGAAGCGGGCAGCA
GTATGCGCAGGCTTGGGGTCGACGAGAGACGACTCCCTCATACTCGGTTACCTCGAGCAATACAATCAATCGAAGCTGCGC

GAATCTCGGCTTGTAAGGGTCGGAAAGGAACCTCGGAGATGGCCACGTCACATCACCAACTTATCGATCTCAGCCGACGTC
GCAGAGAGGGCGAGCGAAGCGGTGAAGGAGGGAAACAATCCCTCGAGAGCATGATCCGTCTGAATCTGCAGCGCAGGAAGC
CGTCACACGCCCGCCTCGAGCGCAGGTCGGGTCCAGCCGGGGGACGAAACGCGCGAGGGCTGATTTCGTGAGCGAAGGAAG
CCGCATCGACAAGTTCGCGTCCCTTTGCCCTCTTTCCCATCACCCGCTCTCGCTCTACCCGCTCAGAACAACACCAGATCA
GTCACAGATATCATGGGCCAGCAGGCGACGCCCGAGGAGCTATACACACGCTCAGAGATCTCCAAGATCAAATTCGCACCC
TTTGGCGTCCCGCGGTCGCGCCGGCTGCAGACCTTCTCCGTCTTTGCCTGGACGACGGCACTGCCCATCCTACTCGGCGTC
TTCTTCCTCCTCTGCTCGTTCCCACCGCTCTGGCCGGCTGTCATTGCCTACCTCACCTGGGTCTTTTTCATTGACCAGGCG
CCGATTCACGGTGGACGGGCGCAGTCTTGGCTGCGGAAGAGTCGGATATGGGTCTGGTTTGCAGGATACTATCCCGTCAGC
TTGATCAAGAGCGCCGACTTGCCGCCTGACCGGAAGTACGTCTTTGGCTACCACCCGCACGGCGTCATAGGCATGGGCGCC
ATCGCCAACTTCGCGACCGACGCAACCGGCTTCTCGACACTCTTCCCCGGCTTGAACCCTCACCTCCTCACCATGAGCACA
GTGCGACGGCGCAACCAGCCAAAGCGCAACGCCTCAGCGCCCGCCAACCAGCTCGATACCGACGATTCCTCGCCCGCTCCC
TCTCGACCTGACTCGCCCGATGTTGACAAAGCGCCAGTGAGGGAGGTCCCGCTGTTCAAGCGGAGGCTGAAGAAGGGGGAT
GGCAAGGTGGAAGACCCGAAGGTCATTCTGGACGAGAAGACGGGCAGGGTTTCTCTCGACTTTCCAAGGACGCCCTACGAC
CAGCTCGACCTGAGCGATTCGTTCCTCGCCGCCTTGCACGAGGCACCCTTCGAGCCGAAGAAGACGTGGACAAAGCGGCGG
AGAGGCTGGTTCTTCTTGGGAGGATTGCTCGGTCTCTGCGCAGGCTGGATGTTCACCGAGGGCGACCCACTCGCCTCTCTC
GCCAATCTCGACCTCGATGCCTTCTCATCCTGGGATCTTCAGTCAATCCTCGCCGACATGCCTTCCCTTTCCGCTCTCAAC
GGGGTCGCCCTCGGTGAACATCCAGCCTGCGCAGAGACCGAGCAATCCTCCCAAGAAGAACCAGCCTCTCCGCCGCTTTGT
CCACGTCTTCTTCGGCTCGAAGGGTGCCTCGTGCAAGGCGGCGAGGAACGAATCGCTCAGGTCGAGCTGGTCGTAGGGCGT
CCTTGGAAAGTCGAGAGAAACCCTGCCCGTCTTCTCGTCCAGAATGACCTTCGGGTCTTCCACCTTGCCATCCCCCTTCTT
CAGCCTCCGCTTGAACAGCGGGACCTCCCTCACTGGCGCTTTGTCAACATCGGGCGAGTCAGGTCGAGAGGGAGCGGGCGA
GGAATCGTCGGTATCGAGCTGGTTGGCGGGCGCTGAGGCGTTGCGCTTTGGCTGGTTGCGCCGTCGCACTGTGCTCATGGT
GAGGAGGTGAGGGTTCAAGCCGGGGAAGAGTGTCGAGAAGCCGGTTGCGTCGGTCGCGAAGTTGGCGATGGCGCCCATGCC
TATGACGCCGTGCGGGTGGTAGCCAAAGACGTACTTCCGGTCAGGCGGCAAGTCGGCGCTCTTGATCAAGCTGACGGGATA
GTATCCTGCAAACCAGACCCATATCCGACTCTTCCGCAGCCAAGACTGCGCCCGTCCACCGTGAATCGGCGCCTGGTCAAT
GAAAAAGACCCAGGTGAGGTAGGCAATGACAGCCGGCCAGAGCGGTGGGAACGAGCAGAGGAGGAAGAAGACGCCGAGTAG
GATGGGCAGTGCCGTCGTCCAGGCAAAGACGGAGAAGGTCTGCAGCCGGCGCGACCGCGGGACGCCAAAGGGTGCGAATTT
GATCTTGGAGATCTCTGAGCGTGTGTATAGCTCCTCGGGCGTCGCCTGCTGGCCCATACTAGTACGATTCCGCCCCGTCTC
ACCTCGCATCCGACCTGTGTAGCCACGCCTTTTCTCTTTCCGCCCCCGACACTAAAAGGAGTTCAGTCGTCCGCTTTTCCT
CTCTTCCGTTCTCCACGCCTGCAGTACTGCATGCCTCAGCTGTGTAATTTCGATAGAGTACTCGCACGTATGCTCGCGCGG
ACTTGTCGAGAGAGCGGGCGAGAGGTCGAGAGGAGCTGACTTGAGTGAGCAGCGAGAGCGGAGCGGCTTGTTGCGGCACAC
AACGGTCCGGCTTACGGACAATCAGCGATGAAGCCGGAAGAGCGGCAGGAGTGGACGAAGACGTGCGAAGAGGAAAGGGGG
CGAGGAAGAGAAGCGGAGGAGGAAACGCAGCGAAGCAGCACAACTTCCGCAGAGACGACGGTTCACATAGTCAAAGATGCA
GTGCAGAGAAGTGCGCGCCGCCTTCAGTTTAAACTATCAGTGTTTGACAGGATATATTGGCGGGTAAAC

(3) ARE1i expression cassette sequence:

TGGCTGGTGGCAGGATATATTGTGGTGTAAACAAATTGACGCTTAGACAACTTAATAACACATTGCGGACGTTTTTAATGT
ACTGAATTAACGCCGAATTGAATTCGAGCTCGGTACCCGGGCCAGACGGACCTTGAGAACCCTCAATCGCTCGCGGTACTC
GTCCGCCCTGCGATCCAGCATCGAAACCGAGTGCAGCGCGTTCAACAAATCCGAGTCGTCTCCTCCTGCTCCTTCGCGCTG
TTTCGGCGCGGGTGGCGCAGGGACAGCCGGAGGGAGAGGGGGAGGAGGAGGAGGGGCTGGGGCGACCTTTTTCTTCTTCTT
CCTGTTCTTGCCCTTCTTCTTCGCCGCCTCTGCTTCTCCATCACCCACCGCCCCCACATTCGCCGCCGAAGCACCGACCAC
GGCCCCCCTCATCCGCCTCTGCAACCTCCTCGCCTCGCTCGCCTCCAAACTCAATCGCGCGACGCACTGCTCCAACTCGGC
GATGGCGCTCATCAAGCTTGGGAGGGAGGCGGGTGAGAGAGCCGAGTCGGAGAGGATGCCGTCTGCGGGGATTTGGGAGGG
TGAGAGGTGGGTTTGAGGCGGTTGAGAGGAGGAGGACGAGAGGGGGAGGGCGGAGGAGAGGGCTGTCAAGTCCGAGAGGGA
GAGGGGGATCGAGGTCGTGAAGGATGTCGAGGACGAGGAAGAGGCGTTCCGCTGTTGTTGTTGCTGGACGGCGGAGAGGAC
GCCCAGGAAGGCTGCGTCGGCCGCTTGCGAGGGCGAGTCGATCACCTCGTAGGCGGCGTCGTCGTGCTCGGACGGCGACGA
CGCGTGGTCCTCCGACTCGACCCAGTCTGGCCCGTTGAAGAGGCATTCGGCGGCGAAGACGACGGCCGGGTCGTCCGAGGC
TGTCGAGGCAAGTGGCTGTAAGCAAGCGCGGCAGAAGAGGCAGAACGCTGCGACGCACTCCTAGGATCCCACGGCTGCGCA
TCCGGATCCTCCCCGCGCTGCTGGCGCCTCGCCCGCTCCCGCTCGTACTCCGCCATGACCGTCAACCCCTGGCAGAGCAGG
CGGTACTGCTCGACCAGGGCCCAGTCCTCGCTCGTCTGTGGGAAAGGTTGGACGGGACATCCTGGCGGCAATGGAAGCGAG
TACAGCCAGTCGAGCGTGAGAGGGGAGGGCCGAAAGTGCTCCGTCGAGATGCTGGCGAGCGAGACGAGGGGCTGGGTGAGC
GACGGCGGGCGGATCCCTGCGCCTGTGTTGTCCATCCCTGCAGTGCACTCTGTTGCTCGTATCATGTCCCACTCCCTTGTA
TCCCTCGAGTCGGTCGACTCTTCCCTGGCGAGTCCAAGCGGAGGAGGTGGTCGTCGCCTGACCCGCTCGGAGTGCGCCGCT
CGACTTGGCCCTGGGAGAACAAGCCTGTGTGAGTCTGTCTAGCCTGTCAGCGAATGCGCCAGACGAGTGCAAGCGGGTGAG
CGAGGTCGACCCTGCTCGTCACTCGCTCGTCGGGTGCGGCCGCATCGTTGAACTTGCACTTCTCACTCGCACTCGCTCTGG
TACAGCTACAGTCACTCGCTTACTACTCTGCAGGTTCACAGCAACTCACCCGTCCAACTCCCACCCTCCCCCGTGCAGCCC
ACCATGGGTACCACTCTTGACGACACGGCTTACCGGTACCGCACCAGTGTCCCGGGGGACGCCGAGGCCATCGAGGCACTG
GATGGGTCCTTCACCACCGACACCGTCTTCCGCGTCACCGCCACCGGGGACGGCTTCACCCTGCGGGAGGTGCCGGTGGAC

CCGCCCCTGACCAAGGTGTTCCCCGACGACGAATCGGACGACGAATCGGACGACGGGGAGGACGGCGACCCGGACTCCCGG
ACGTTCGTCGCGTACGGGGACGACGGCGACCTGGCGGGCTTCGTGGTCGTCTCGTACTCCGGCTGGAACCGCCGGCTGACC
GTCGAGGACATCGAGGTCGCCCCGGAGCACCGGGGGCACGGGGTCGGGCGCGCGTTGATGGGGCTCGCGACGGAGTTCGCA
CGCGAGCGGGGCGCCGGGCACCTCTGGCTGGAGGTCACCAACGTCAACGCACCGGCGATCCACGCGTACCGGCGGATGGGG
TTCACCCTCTGCGGCCTGGACACCGCCCTGTACGACGGCACCGCCTCGGACGGCGAGCAGGCGCTCTACATGAGCATGCCC
TGCCCCTAGGATCGTTCAAACATTTGGCAATAAAGTTTCTTAAGATTGAATCCTGTTGCCGGTCTTGCGATGATTATCATA
TAATTTCTGTTGAATTACGTTAAGCATGTAATAATTAACATGTAATGCATGACGTTATTTATGAGATGGGTTTTTATGATT
AGAGTCCCGCAATTATACATTTAATACGCGATAGAAAACAAAATATAGCGCGCAAACTAGGATAAATTATCGCGCGCGGTG
TCATCTATGTTACTAGATCGGGCCTGGATCCTCTAGATCCATGCTGCTGCGATCTGGGAGTGCAAGCCCGCGGTCAGCAGC
CATTGCCCGCCTACAATGTCTTCCCAAAGCCGCCCTTGCTCGCTCGCTGCGTGGAGTTCGACGTTCTCCTCGCTCCGCAAG
CATTGGAATGAACCTTGCTCTCTAGTTCCCTCCTCCGTGACCTCGTTTCGTCCTTTAGACGGCACGATGGAAGGAAGAAAT
CTCTGCGGACAAGCAAATCTGCTGGCTCGCCTTGTAGGTCGCCTACCGGAGCAAGCCTTGTGCCGCCGGGATGCCAACGTC
GTTTTTTGACGTTTGCAAGACGTAGAGGACGCTTCGGACGACGAAACAAGCTGTGAGGACATGGAAGTCGTGGGAGGAACG
GCGCAGAGCGGCGCCGCGGGAGCATAAGGCAAGCGAGATAGTCCAGAAATCGCGGCGCCAAGTACAGTAATTTATTGGAGC
AGGCACCAGAAGCGGGCAGCAGTATGCGCAGGCTTGGGGTCGACGAGAGACGACTCCCTCATACTCGGTTACCTCGAGCAA
TACAATCAATCGAAGCTGCGCGAATCTCGGCTTGTAAGGGTCGGAAAGGAACCTCGGAGATGGCCACGTCACATCACCAAC
TTATCGATCTCAGCCGACGTCGCAGAGAGGGCGAGCGAAGCGGTGAAGGAGGGAAACAATCCCTCGAGAGCATGATCCGTC
TGAATCTGCAGCGCAGGAAGCCGTCACACGCCCGCCTCGAGCGCAGGTCGGGTCCAGCCGGGGGACGAAACGCGCGAGGGC
TGATTTCGTGAGCGAAGGAAGCCGCATCGACAAGTTCGCGTCCCTTTGCCCTCTTTCCCATCACCCGCTCTCGCTCTACCC
GCTCAGAACAACACCAGATCAGTCACAGATATCATGTCGACTGTTACCGGCGAACTCGATTACAAAGAGAGCCATCACATT
CGCACTGCAATCGACAGTAATCGACCCTGCGTGTATGACGGAGCGATCCCTTCCAGTGACGCTCCCTCTTCCTCGAAACTT
TGCGCTCACACCGCACCAGATGGCCTCGCCAGACCCGCCACTCCCAGGCCCAGCCAACCTCGTCGACGACGCACTCCGACA
CCCAGACTCGGCGCCGCCCATCTCGCCCGACTCCGCGCCTCCTTCGACTGCGACTCGGCCCTCTGCTCTCTCGCGCGGAGA
GCTCTCGACCGCTTCGAGCTACGCGAGCGAGGTGTCGACGAGGGAGGGGACACCGGATCTGGCGAATGGGTGAGTAGGCGC
GAGAACAAGTCCCGAGGTCTTGATCAGTGGGGGAGGTGTGAGCGGCGAGGGAGCGAGATGCAGGGACTGACTGGCTGCGCG
CGTGCAGGCAAGGGGTTACGACGACCATCACGACTGTCACAGGCAAAGGCGGAAAGGCCGTCACCCAGACGTGCGTTTCCA
GACTCCTCCCTCTCCTCGATGCTTCCTTCTTGCACGAAGTCTGGGAGATTGACGCTGTTCTCTCCGTTTGCCTGTGACAGT
CGTGATGGTCGTCGTAACCCCTTGCCTGCACGCGCGCAGCCAGTCAGTCCCTGCATCTCGCTCCCTCGCCGCTCACACCTC
CCCCACTGATCAAGACCTCGGGACTTGTTCTCGCGCCTACTCACCCATTCGCCAGATCCGGTGTCCCCTCCCTCGTCGACA
CCTCGCTCGCGTAGCTCGAAGCGGTCGAGAGCTCTCCGCGCGAGAGAGCAGAGGGCCGAGTCGCAGTCGAAGGAGGCGCGG
AGTCGGGCGAGATGGGCGGCGCCGAGTCTGGGTGTCGGAGTGCGTCGTCGACGAGGTTGGCTGGGCCTGGGAGTGGCGGGT
CTGGCGAGGCCATCTGGTGCGGTGTGAGCGCAAAGTTTCGAGGAAGAGGGAGCGTCACTGGAAGGGATCGCTCCGTCATAC
ACGCAGGGTCGATTACTGTCGATTGCAGTGCGAATGTGATGGCTCTCTTTGTAATCGAGTTCGCCGGTAACAGTCGACATA
CTAGTACGATTCCGCCCCGTCTCACCTCGCATCCGACCTGTGTAGCCACGCCTTTTCTCTTTCCGCCCCCGACACTAAAAG
GAGTTCAGTCGTCCGCTTTTCCTCTCTTCCGTTCTCCACGCCTGCAGTACTGCATGCCTCAGCTGTGTAATTTCGATAGAG
TACTCGCACGTATGCTCGCGCGGACTTGTCGAGAGAGCGGGCGAGAGGTCGAGAGGAGCTGACTTGAGTGAGCAGCGAGAG
CGGAGCGGCTTGTTGCGGCACACAACGGTCCGGCTTACGGACAATCAGCGATGAAGCCGGAAGAGCGGCAGGAGTGGACGA
AGACGTGCGAAGAGGAAAGGGGGCGAGGAAGAGAAGCGGAGGAGGAAACGCAGCGAAGCAGCACAACTTCCGCAGAGACGA
CGGTTCACATAGTCAAAGATGCAGTGCAGAGAAGTGCGCGCCGCCTTCAGTTTAAACTATCAGTGTTTGACAGGATATATT
GGCGGGTAAAC

(4) TGL5-LDP1 expression cassette sequence:

TGGCTGGTGGCAGGATATATTGTGGTGTAAACAAATTGACGCTTAGACAACTTAATAACACATTGCGGACGTTTTTAATGT
ACTGAATTAACGCCGAATTGAATTCGAGCTCGGTACCCGGGCCAGACGGACCTTGAGAACCCTCAATCGCTCGCGGTACTC
GTCCGCCCTGCGATCCAGCATCGAAACCGAGTGCAGCGCGTTCAACAAATCCGAGTCGTCTCCTCCTGCTCCTTCGCGCTG
TTTCGGCGCGGGTGGCGCAGGGACAGCCGGAGGGAGAGGGGGAGGAGGAGGAGGGGCTGGGGCGACCTTTTTCTTCTTCTT
CCTGTTCTTGCCCTTCTTCTTCGCCGCCTCTGCTTCTCCATCACCCACCGCCCCCACATTCGCCGCCGAAGCACCGACCAC
GGCCCCCCTCATCCGCCTCTGCAACCTCCTCGCCTCGCTCGCCTCCAAACTCAATCGCGCGACGCACTGCTCCAACTCGGC
GATGGCGCTCATCAAGCTTGGGAGGGAGGCGGGTGAGAGAGCCGAGTCGGAGAGGATGCCGTCTGCGGGGATTTGGGAGGG
TGAGAGGTGGGTTTGAGGCGGTTGAGAGGAGGAGGACGAGAGGGGGAGGGCGGAGGAGAGGGCTGTCAAGTCCGAGAGGGA
GAGGGGGATCGAGGTCGTGAAGGATGTCGAGGACGAGGAAGAGGCGTTCCGCTGTTGTTGTTGCTGGACGGCGGAGAGGAC
GCCCAGGAAGGCTGCGTCGGCCGCTTGCGAGGGCGAGTCGATCACCTCGTAGGCGGCGTCGTCGTGCTCGGACGGCGACGA
CGCGTGGTCCTCCGACTCGACCCAGTCTGGCCCGTTGAAGAGGCATTCGGCGGCGAAGACGACGGCCGGGTCGTCCGAGGC
TGTCGAGGCAAGTGGCTGTAAGCAAGCGCGGCAGAAGAGGCAGAACGCTGCGACGCACTCCTAGGATCCCACGGCTGCGCA
TCCGGATCCTCCCCGCGCTGCTGGCGCCTCGCCCGCTCCCGCTCGTACTCCGCCATGACCGTCAACCCCTGGCAGAGCAGG
CGGTACTGCTCGACCAGGGCCCAGTCCTCGCTCGTCTGTGGGAAAGGTTGGACGGGACATCCTGGCGGCAATGGAAGCGAG

TACAGCCAGTCGAGCGTGAGAGGGGAGGGCCGAAAGTGCTCCGTCGAGATGCTGGCGAGCGAGACGAGGGGCTGGGTGAGC
GACGGCGGGCGGATCCCTGCGCCTGTGTTGTCCATCCCTGCAGTGCACTCTGTTGCTCGTATCATGTCCCACTCCCTTGTA
TCCCTCGAGTCGGTCGACTCTTCCCTGGCGAGTCCAAGCGGAGGAGGTGGTCGTCGCCTGACCCGCTCGGAGTGCGCCGCT
CGACTTGGCCCTGGGAGAACAAGCCTGTGTGAGTCTGTCTAGCCTGTCAGCGAATGCGCCAGACGAGTGCAAGCGGGTGAG
CGAGGTCGACCCTGCTCGTCACTCGCTCGTCGGGTGCGGCCGCATCGTTGAACTTGCACTTCTCACTCGCACTCGCTCTGG
TACAGCTACAGTCACTCGCTTACTACTCTGCAGGTTCACAGCAACTCACCCGTCCAACTCCCACCCTCCCCCGTGCAGCCC
ACCATGGAGGCCGACTTGTCCCGCCCGCCTCCCGCTCCCTCATCCTCCAGATCTCGACCCCCACAACCCTCTCGCCGACCA
ACCTCCGCCCAAACCTACACCGTCACGCAGCTCGAACAGAGTCGGGGGAAGGGATGGGACAAGTGGAAGGAGTGGGACGAG
CTCGCGACGGATTTGGGGTGCGATTGGGTTGTTGAAGGCGATATGGAGGCGTTTGGAAAGGTGAGTTCTGCCGTGCGGTGC
GCTCGCTGGGAAGGCCAGGCTGCGTTGATGAGGTGTCAATGAGGGAGGGGACGGCTCGCGCGAGGCGGCTTGCAAGACGCG
GATCTTGGTTGTCCTCGTCGAAGCGGTCTTTCAGGCGGATCACGGCTCAACCAAGTCGTTCGACGGTACTGACACCCTCGA
CCACGCTCACAGGCTCTATCAGAACCTCCGGCAGACGACCAGCCTGAGCGTATCATCGCAGCGTCCGACTGGGCGCCCGTC
AAGGAGCAGAAACGACGAAGGTGCGTCGGGCAACCTGCTCTCTCAGCTCGCCAAGTGCCTATCCACAGCACAAGAGACTGA
GGGATCTTGTTGTCCTGCCTGTGCAGAGGACCGAAGAAGCGGAAAGACGTTGTGCGAGAAGGCTGGGCCTACCACATCTCT
CGCTGGCCACTCCTCGTGCGTCCGGCTCCTCTCTTACCATTCCCGTGCGCTTCCTGCGCTTCTGAGCGAAGGAACTGATAG
AGGTTGACTTGTGACGTGCAGATGCTCATCTTCACGATCATCTTCCTCGAGTTTGTCGGCTACCTCTTCGTCCGGCAATGC
GTCAACGTGATCGAGTTCTTCGCGGGATGTGCGTCGACCCTCGTTCCACTCGGTCGTGATTCCCCGTGAGGGCAGCAGAAA
CTGATAGAAAGCGAGGATCTCGATGCAGGGCGAGGCGAGCGAGGACGCCTCCGACTAGCGTTGCGGAACGCTAAGACGTAC
GAAGAGGTGCGCCCGCTCTCCTGCTTTCAACAGAGAAGCAGAACACACGATGCCTGTAGCTGATGCATCCGTTGTCCGGCT
TCGCAGTGGAAGCAACGCGCCCTCGCCCTCGACTCCTTCCTCGGCCTGTCGCACTGGAAGTCCTCCCCTCCGAACGCCTAT
TACGACTCCGCCCTGATCCGCCGTGTCCTGCGCTCCCTGCGCGACCTCCGCACTCGCGACGATGCCGAAGGGGTCTGCGCT
GTCCTGCACACGTGCGTGAGGAACAACTTTGCGGGAGTCGAGAGCTTCAGGTTGTATAGCGAGAGCTATTACGGGACGAAA
GAGCTCGTTCAAGAGTTTATCGACGAGGGTGCGTTTCCAATACCTTCTCGCTCGGCTGCATCGAGCTGACCTTCGCGAACT
TCACGCAGTGACCCGTTCGCTCGAATACATCCGCCAAGTGCCGCCTTCGCTCTTGCCAGTCGAGGAGAAGGCAGACTTCTT
CCGCTCGGTTGCGAAGAACCTGGGCGCGTCGGCTCTGTGTCTGTCGGGAGGTGCCTGTGAGTTGCGCGGCAATGGCTTGGT
CTTCCACATCCTTCCTCGAAGTGTGTCGCTTATCAAGGGCTTGTCTCGGTCCCACAGCTTTCGGCTAGTACGCCTTACGAC
TCCGCCATCGAGACGAGACGAGGACTGACGAGCCGGTCCCTGCTTGCAGCTACCACTTCGGCGTCATCCGCGCCCTGCTCG
ATCAAAATCTGCTACCGCGGGTCATCACCGGTACTTCCGCCGGCGCAATCAGTGCGTTCACATCTTGCCATCCTCCCCTCC
GTCGTAGCTGACGAATACACCCTGCAGTCGCCGCCTTCTGCTGCACCCGCACCGACGCCGAACTCCGCCAACTCATCACTC
CAACTCTCGCAGACCGCATCACAGCTTGCGAAGAGCCCTTCCGAGTTTGGGCGAAGCGGGCGTGGAAGACGGGCGCGAGGT
TCGATACGGTCCAGTGGGCGAGGAAGGCGAGCTTCTTCACGATGGGAAACATGACGTTCAAGGAGGCGTTTGAGCGGACGG
GCAGGATCTTGAACGTCAGCGGTGCGTCCGCAGACTACTGTGGTCACTCCACTTCCTGCTCTCTAGCAACCGACTGACTCG
TCCGACTGTCGTACAGTCATCCCCGCCGACACTCACTCGCCGACCAAGCTCCTCAACTACCTCACCGCGCCCGACTGCGTC
ATCTACTCCGCCGTCATCGCCAGCGCCGCCGTCCCAGGCATCATCAACCCGGTCGTCTTGATGACGAAGGGCAAGGACGGC
AAGCTCCGCCCGCACGATTTTTCGCAGCTGCACAAGGACGGCTCGTTGCGGGTCGATATTCCTCTACGTGAGTCGGACAGA
CGTGGCCTGATTGCGCTCGTTAGCGCTGCGAGGCTGACCTGCCGCCACGCCTTCGCCTCGCAGAGGACCTCCACCTCCTGT
ACAACGTCAACTTCAGCGTCGTCTCGCAAGTGAATCCGCACATCCACCTCTTCAACTTTGCTTCACGCGGCTCACCAGGCC
GACCGGTTTCGCATCGACGGGGACGAGGATGGAGGGGCGGGTGGTTCCTCAGCGCGGCCGAGCACTACCTCAAGATTGAGA
TGCTCAAGTGGTTCAGGGTGCGTTGCGCTGTCGGATTTCGTTCATTACCTGCTTAGGTATTCGAGCAAGCTACTGATTAGA
GATCCCAACCCCGTGTGCAGGTCATCCGCGACCTTGAGCTTCTCCCCGAACTCGGCAACCAGAACTGGAGCAACGTCTTCC
TGCAAAAGTTCGAGGGCAGCGTGACCATCTGGCCGAAGAGCAGGAGTGCGTCCGCTTCTACTGTCTCAAAAGGCTCTACTT
GGCTTGCTAACCTGAATCCGCTACTGCAGTCGCCGACTGGCCTCGCATCCTGACGGACCCGGACAGGAACGAGCTGGCGAG
GATGATCCGCGTTGGACAGCAAGTCACTTGGCCTAAAATCAAGATGATCGAGAACCGCCTGAAGTGCGTTTCCTCCGGCTT
GTCGTCCTCGTGCTGCTGCTTACGACCGACTCGTTTGCAGAATCGAACGCCAGATCGACCTTGGCCGCTCTGAAGTCCGCC
GCGCGTTCAACGACTCCGACGCGACCTCCCACTCTCTCGCTCCGCCCACACTCAGCCGCCAAGCCTCTTCGGGCGAGATCA
TGACTTCAGCACGTCCTCGAGCGACGAACGATCGCTCGAGCCCCGAGGAGAACGGTCGGGGCAGCCGTCGCAACTTCCGCG
CCGTCGACATGGAAGCGTCGTCATCGTCCGGCGGCGATCCCGTCTCAACCTCGAACGGCGGCGAAGAGACGCCTGCGAAGC
GTCGACAGGCAATCCTCGCTCGACTCGGTCTCAAGGGCGCGGCTGGGAAGGGTTCGCGGGATCTCTATGGCGGGACGGTCG
AGAGCGGAGGCGAGGAGAAGTACACGGACGGCGAGAGCGACTGGACAAGCGGAGGAGAGGGGACGTCGTATGCCGGTGTTG
AGCGAGGGTCGTCTTCGGGCAAGATGCGGAAGAGGCGAGCGAGTATGAGCAGCTTTGGCAGGCGGCCGAGGCGGGATACTC
TCTCGAGCGACGAGGATCCCTGGCACGGCGGCGGCTCGGCCACCGTCAACGAGAAGCAGCCCGCCACCGACGCGCCCCTCG
CGCACGAGACCGCCATCCACCGCGTGCGTCCCCATCTCTTCCTCGTGAAAACCCGCTCAACAACTCACACACGTGCAACAC
AGGCGCTCGACTACCCCGTGATCAAAGACACCCTCTCGACCTTTGACCACTACGCCCACTCGCACCCCTACATCTCGTCCC
TCTACTCGCGCACCCTCAGCCTCTCGCGCCAGATCCTCGCTCACGTCCAGCCCGTCCTCCCCCTCGAGTTGGCGGACCAGT
ACGCGAACAAGACGCTCGATGTCGTCGAGAAGTATGTCCCGCAGGTCAAGATGGAGACGGGAGAGTTGATTGGAAAGGCGA
GGGGGCCCGCCGATGCCGCGTTCCAGACGGCCCAGGAGTACAGGCAGGGAATCCAGTCTGTGAGTCGCGCGCCGGCGTTTT
CTCTCCTTCTTTGTCGCTCGTTCTCGCCGAGCGCGAGGGCGGTCGACTGCTTCAGGGAGAGCGCTGTCGCTGACATTTCTC
CCTTCTCCCTTGTGCGCGTCCCGCAGCGCATCTCGCCCGTGACGGACCAGCTCTACCAGCGCATCACGACCTCGCAAGCGC
ACCTCTCGTCCCTCCAAGACCGTCTCCAAAAGACGATCAAGCAGCGTGCGTCCCTCTCCCTCCCTTTCCCTTCCTCCCTTC

TCCCGCTGACAGGCTTGAGTGGATGCGATGCAGTCCCCCACGACACGGAGAGCCTCCAGTCTACGCTCCACTCGATCCTCA
ACGAGGTCGACGGGTTGGTCAAGTCTGCTCAGTCGATCGTGCGTCCTTCCCTCCCCTCCCTCCCTTCTTCCGACCGACGAG
CTGACGATAGGATTTGTGGGGATGTGATAAAGCCCGCCAACGCCCAAGCGACCGCCAAGCCCGTCTTTGACGGAGTCGTCG
AGGCTGCGGACCACATTCGCAAGGAGGTCACTCGCACCGGTGCGTCCTCCCGCGCTCTCTCCTCTCTTCTCGTCGACTTTG
ACGAGATACCGAGAAAGTTCAGCAAGACGACGCTGACTCTCTGCCCTCGCTGCGCTCGCGCAAACAGACATCCCGATGGGC
GCCCGCGCGCAAAACGTCCTCACCTACACCCAGGACCGCCTCCAGCCCGTCGTCGAGCAGATCAAGAGTTTCGTGCTCAAG
AAGAAGGACGAGGTTGCCGAGACTGTCGAGGAGAAGGAGGGCGAGGGGGAGAAGCAGCACCACCATCACCATCACGGCTCG
GGCGTCAAGCAGACCCTCAACTTCGACCTCCTCAAGCTCGCTGGCGACGTCGAGTCGAACCCTGGCCCTATGGCCAAGTTG
ACCAGTGCCGTTCCGGTGCTCACCGCGCGCGACGTCGCCGGAGCGGTCGAGTTCTGGACCGACCGGCTCGGGTTCTCCCGG
GACTTCGTGGAGGACGACTTCGCCGGTGTGGTCCGGGACGACGTGACCCTGTTCATCAGCGCGGTCCAGGACCAGGTGGTG
CCGGACAACACCCTGGCCTGGGTGTGGGTGCGCGGCCTGGACGAGCTGTACGCCGAGTGGTCGGAGGTCGTGTCCACGAAC
TTCCGGGACGCCTCCGGGCCGGCCATGACCGAGATCGGCGAGCAGCCGTGGGGGCGGGAGTTCGCCCTGCGCGACCCGGCC
GGCAACTGCGTGCACTTCGTGGCCGAGGAGCAGGACTAGACGATTCCGCCCCGTCTCACCTCGCATCCGACCTGTGTAGCC
ACGCCTTTTCTCTTTCCGCCCCCGACACTAAAAGGAGTTCAGTCGTCCGCTTTTCCTCTCTTCCGTTCTCCACGCCTGCAG
TACTGCATGCCTCAGCTGTGTAATTTCGATAGAGTACTCGCACGTATGCTCGCGCGGACTTGTCGAGAGAGCGGGCGAGAG
GTCGAGAGGAGCTGACTTGAGTGAGCAGCGAGAGCGGAGCGGCTTGTTGCGGCACACAACGGTCCGGCTTACGGACAATCA
GCGATGAAGCCGGAAGAGCGGCAGGAGTGGACGAAGACGTGCGAAGAGGAAAGGGGGCGAGGAAGAGAAGCGGAGGAGGAA
ACGCAGCGAAGCAGCACAACTTCCGCAGAGACGACGGTTCACATAGTCAAAGATGCAGTGCAGAGAAGTGCGCGGGATCCT
CTAGAGTCGACCTGCAGCATGCAAGCTTGGAGCTTGAGCTTGGATCAGATTGTCGTTTCCGCCTTCAGTTTAAACTATCAG
TGTTTGACAGGATATATTGGCGGGTAAAC
